# Multi‐Species Telemetry Quantifies Current and Future Efficacy of a Remote Marine Protected Area

**DOI:** 10.1111/gcb.70138

**Published:** 2025-04-15

**Authors:** Morgan E. Gilmour, Kydd Pollock, Josh Adams, Barbara A. Block, Jennifer E. Caselle, Alex Filous, Alan M. Friedlander, Edward T. Game, Elliott L. Hazen, Marie Hill, Nick D. Holmes, Kevin D. Lafferty, Sara M. Maxwell, Douglas J. McCauley, Robert Schallert, Scott A. Shaffer, Nicholas H. Wolff, Alex Wegmann

**Affiliations:** ^1^ U.S. Geological Survey Western Ecological Research Center, Santa Cruz Field Station Santa Cruz California USA; ^2^ Earth Science Division National Aeronautics and Space Administration, Ames Research Center Moffett Field California USA; ^3^ The Nature Conservancy Honolulu Hawaii USA; ^4^ Department of Oceans Stanford University Pacific Grove California USA; ^5^ Marine Science Institute University of California Santa Barbara Santa Barbara California USA; ^6^ Pristine Seas National Geographic Society Washington, DC USA; ^7^ Hawaiʻi Institute of Marine Biology University of Hawaiʻi Hawaii USA; ^8^ The Nature Conservancy South Brisbane Queensland Australia; ^9^ Ecosystem Science Division Southwest Fisheries Science Center, National Oceanic and Atmospheric Administration Monterey California USA; ^10^ Pacific Islands Fisheries Science Center National Oceanic and Atmospheric Administration Honolulu Hawaii USA; ^11^ The Nature Conservancy Sacramento California USA; ^12^ U.S. Geological Survey, Western Ecological Research Center, Santa Barbara Field Station c/o Marine Science Institute University of California Santa Barbara Santa Barbara California USA; ^13^ School of Interdisciplinary Arts and Sciences University of Washington Bothell WA USA; ^14^ Department of Biological Sciences San Jose State University San Jose California USA; ^15^ The Nature Conservancy Brunswick Maine USA

**Keywords:** CMIP6, GPS‐tracking, marine spatial planning, movement ecology, pseudo‐absence, satellite‐tracking

## Abstract

Large‐scale marine protected areas (LSMPAs; > 1000 km^2^) provide important refuge for large mobile species, but most do not encompass species' ranges. To better understand current and future LSMPA value, we concurrently tracked nine species (seabirds, cetaceans, pelagic fishes, manta rays, reef sharks) at Palmyra Atoll and Kingman Reef (PKMPA) in the U.S. Pacific Islands Heritage Marine National Monument. PKMPA and the U.S. Exclusive Economic Zone encompassed 39% and 54% of species movements (*n* = 83; tracking duration range: 0.5–350 days), respectively. Species distribution models indicated 73% of PKMPA contained highly suitable habitat. Under two projected future scenarios (SSP 1–2.6, “Sustainability”; SSP 3–7.0, “Rocky Road”), strong sea surface temperature gradients initially could cause abrupt oceanic change resulting in predicted habitat loss in 2040–2050, followed by an equilibrium response and regained habitat by 2090–2100. Current and future suitable habitats were available adjacent to PKMPA, suggesting that increased MPA size could enhance protection. Our three‐tiered approach combining animal tracking with publicly available remote sensing data and future projected environmental scenarios could be used to design, study, and monitor protected areas throughout the world. Holistic approaches that encompass diverse species and habitat use can enhance assessments of protected area designs. Animal telemetry and remote sensing may be helpful for ascertaining the extent to which other MPAs protect large mobile species in the future.

## Graphical abstract

We combined animal telemetry, remote sensing, and projected future environmental scenarios to assess how nine species used a remote marine protected area (MPA) in the Pacific Ocean under current and future conditions. Seabirds, cetaceans, tuna, manta rays, and reef sharks used habitats on both sides of the MPA boundaries and were predicted to use habitats inside and adjacent to the MPA in the future. Our three‐tiered, holistic approach could help design, study, and monitor MPAs around the world.

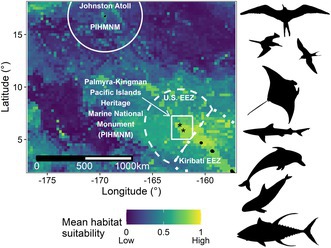

## 1 Introduction

Although seabirds, fishes, and cetaceans do not recognize jurisdictional boundaries, crossing them can increase mortality risk. Thus, marine protected areas (MPAs), which offer one potential solution to combat global declines in biodiversity, may not protect species equally: mobile marine animals are unlikely to stay within even very large MPAs (> 150,000 km^2^ Conners et al. 2022) and ephemeral ocean processes on which these species rely may only occur within an MPA for part of the year (e.g., Gilmour et al. 2022). Additionally, MPAs are subject to external threats like seabed mining (Amon et al. 2023), illegal fishing (White et al. 2017), and passive fishing strategies (drifting fish aggregation devices; dFADs; Curnick, Feary, et al. 2020). These cumulative threats increase risk to biodiversity, especially within tropical marine ecosystems, where animals connect coral reefs to adjacent terrestrial and pelagic ecosystems (e.g., McCauley, DeSalles, et al. 2012). Climate change creates additional risk and is expected to affect tropical marine ecosystems inconsistently (e.g., predicted changes in temperature and rainfall variably affect physical ocean processes like thermoclines and species ranges throughout the global tropics; (e.g., predicted changes in temperature and rainfall variably affect physical ocean processes like thermoclines and species ranges throughout the global tropics; Eddy et al. 2021; H. Kim et al. 2023). To understand the extent that large‐scale MPAs (> 1000 km^2^; LSMPAs) can support biodiversity and conservation now and in the future, we assessed the efficacy of protections provided by the Palmyra Atoll and Kingman Reef unit (PKMPA) of the Pacific Islands Heritage Marine National Monument (PIHMNM).

The massive PIHMNM (1.2 million km^2^) was established in 2009, comprised of five units based on geography (50 nm from centrally located islands or atolls; U.S. Presidential Proclamation 8336 2009). Expansion in 2014 increased the area of three units to the full extent of the U.S. Exclusive Economic Zone (EEZ; 200 nm) to include seamounts, deep‐sea corals, and mobile species' diverse habitats (U.S. Presidential Proclamation 9173 2014). However, the size of the two remaining units (PKMPA; Howland and Baker Islands) was not increased, despite high coral reef and terrestrial biodiversity. In 2023, U.S. President Biden recommended increasing PKMPA's area (Biden 2023). Because of PKMPA's established climate resilience (Fox et al. 2023; Khen et al. 2022) and diverse and abundant highly mobile marine species (Gilmour et al. 2022; White et al. 2017; Young et al. 2015), quantification of MPA efficacy within the context of climate change could inform whether MPA expansion could improve regional conservation outcomes on longer timescales.

Largely due to the cost of accessing and sampling these regions, the conservation benefits of remote LSMPAs that surround atolls are relatively unknown compared with MPAs in coastal environments (Blanluet et al. 2024; Carlisle et al. 2019). Remote LSMPAs could contribute ecosystem resiliency required to support species, resources, and conservation actions affected by climate change (Sala et al. 2021). Given the uncertainty surrounding habitat changes and species adaptations to project future changes in the environment, we integrated animal telemetry data with environmental remote sensing and climate change scenarios to evaluate how PKMPA protects species movements and current and future habitats. We concurrently tracked nine species with diverse habitat requirements: three seabirds (great frigatebird, *Fregata minor* , red‐footed booby, *Sula sula* , and sooty tern, *Onychoprion fuscatus* ), two cetaceans (bottlenose dolphin, *Tursiops truncatus* , and melon‐headed whale, *Peponocephala electra* ), two pelagic fishes (blue marlin, *Makaira nigricans* , and yellowfin tuna, *Thunnus albacares* ), reef manta rays (*Mobula alfredi*), and grey reef sharks ( *Carcharhinus amblyrhynchos* ).

We provide a framework that can be adapted to assess MPA efficacy in other regions; such assessments could help countries meet conservation initiatives like “30 X 30” (Target 3 of the Kunming‐Montreal Global Biodiversity Framework) and United Nations Sustainable Development Goals (Lewis et al. 2017; United Nations 2015). Multifaceted approaches are critical for evaluating ongoing conservation efforts challenged by environmental change. Integrative frameworks can demonstrate how diverse taxa respond to the same local and mesoscale environmental conditions. Furthermore, applying habitat models to climate change scenarios can estimate how these protections could extend into the future. Other studies provide valuable insight to animal movements within, across, and outside protected area boundaries, but these studies are often retrospective (typically encompassing a longer but coarser timeframe that generalizes trends across years (Conners et al. 2022) or focus on related taxa that may only provide perspective on one part of the ecosystem (Carlisle et al. 2019; Filous et al. 2022). Because nearshore and pelagic ocean habitats are inherently linked, it is critical to consider how animals use and move through these habitats to support effective conservation efforts. Our approach can be adopted globally as an example of a decision‐support tool for the maintenance of current protected areas, and the design of future protected areas. The capacity for an MPA to retain high biodiversity by protecting movements and habitats of highly mobile species could help preserve ecosystem services (e.g., nutrient deposition, fisheries spillover) to buffer climate change effects and perpetuate healthy marine ecosystems (Boerder et al. 2019; Carlisle et al. 2019).

## 2 Methods

### 2.1 Data Collection

We deployed electronic satellite and archival tags (GPS, GPS‐Argos, Argos PTT, Argos‐Fastloc, Argos geolocation) on nine species at Palmyra Atoll (5.881973°, −162.075645149°) from 27 February to 04 June 2022 (Table 1). Animal telemetry data are published (Gilmour et al. 2024) and available at: https://doi.org/10.24431/rw1k8ez.

**TABLE 1:** Summary of geolocation and satellite tag tracking datasets and vertical movements of nine species tagged within the Palmyra‐Kingman marine protected area (MPA) and U.S. exclusive economic zone (EEZ). Space use was determined with 50% (core habitat) and 95% (general habitat) kernel density estimates. Dashed lines indicate that species did not have enough data (number of locations) for kernel density estimates. Mean dive depths were calculated from a subset of individuals (yellowfin tuna: 6; bottlenose dolphin: 1; melon‐headed whale: 2).

Primary habitat	Species	No.	Tracking period	Mean (± SD) deployment duration (days)	Mea*n* (± SD) max. distance from Palmyra [km] (max. dist)	Mea*n* (± SD) daily dive depth [m] (max. depth)	Size of area used (km^2^)									
							Inside MPA		Outside MPA		Inside EEZ		Outside EEZ		% Space used (95% UD)	
							50%	95%	50%	95%	50%	95%	50%	95%	Inside MPA	Inside EEZ
Reef–pelagic	Manta ray	4	29 May 2022–25 April 2023	194 ± 115	662 ± 705 (1679)	105 ± 121 (768)	836	3756	32,127	154,230	64,098	232,177	720,421	2,449,432	2.4	8.7
	Grey reef shark	6	30 May 2022–15 May 2023	219 ± 80	169 ± 57 (237)	60 ± 46 (440)	562	2980	741	7194	8012	45,225	6265	43,852	29.3	50.8
Pelagic	Yellowfin tuna	9	29 May – 16 Nov 2022	101 ± 64	336 ± 370 (1256)	124 ± 124 (1160)	1262	5142	38,720	157,042	43,088	197,458	526,221	2,054,766	3.2	8.8
	Blue marlin	1	29 May– 5 August 2022	68	2441	70 ± 59 (352)	30	160	96,376	332,311	1959	10,327	939,680	3,245,027	0.05	0.3
	Sooty tern	12	29 May– 17 June 2022	5 ± 2	312 ± 146 (531)	—	1220	4508	19,500	69,433	95,257	392,601	—	—	6.1	100
	Great frigatebird	7	1 June 2022–29 March 2023	209 ± 122	89 ± 52 (384)	—	567	3038	4517	23,769	6588	45,047	37,587	169,142	11.3	21
Nearshore–pelagic	Bottlenose dolphin	5	30 May–25 June 2022	19 ± 5	30 ± 16 (54)	327 ± 190 (775)	2	16	—	—	2	16	—	—	100	100
	Melon‐headed whale	8	31 May–18 June 2022	13 ± 4	64 ± 58 (194)	281 ± 174 (663)	65	592	—	—	712	6944	—	—	100	100
	Red‐footed booby	31	27 Feb–1 March 2022; 29 May–9 Aug 2022	38 ± 29	49 ± 30 (166)	—	136	1240	—	—	1246	12,286	—	—	100	100

#### 2.1.1 Seabirds

We captured breeding seabirds at nest sites by hand, with hand‐held nets, or with noose poles. At the time of tag deployment, frigatebirds and sooty terns were incubating eggs, and red‐footed boobies were either incubating eggs (*n* = 22) or brooding chicks (*n* = 9). All tracking tags were taped to the central 2–3 tail feathers with waterproof cloth tape (Tesa 4651, Hamburg, Germany). Locations were recorded by either e‐obs Bird Solar 15 g (great frigatebird, *n* = 8, and red‐footed booby, *n* = 8; sample rate: 300 s), i‐gotU GT‐120 (red‐footed booby, *n* = 17; sample rate: 120 s), Lotek Pinpoint50 (red‐footed booby, *n* = 4, and sooty tern, *n* = 4; sample rate: 120 s), Lotek Pinpoint75 (sooty tern, *n* = 8, sample rate: 60 satellite fixes), or TechnoSmart Axy‐Trek mini (red‐footed booby, *n* = 2, sample rate: 120 s). For e‐obs tags, we secured a thin plastic baseplate to 3–4 central tail feathers and attached the tag to the plate with three black nylon cable ties (Panduit PLT1M‐C0, Tinley Park, IL, USA; Adams et al. 2020). We weighed birds to the nearest 5 g, and tag mass did not exceed 2.6% of bird body mass for any individual. Tracking data were either manually downloaded upon tag recovery (i‐gotU, Lotek Pinpoint50, and Technosmart Axy‐Trek tags), downloaded from Argos (Lotek Pinpoint75 tags), or remotely downloaded to two base stations within the atoll, which were recovered approximately monthly (e‐obs tags).

#### 2.1.2 Cetaceans

We deployed satellite (Argos PTT and Argos Fastloc) tracking tags (Wildlife Computers SPLASH‐10, SPLASH10‐F, and SPOT365‐S) on bottlenose dolphins (SPLASH, *n* = 3; SPOT, *n* = 2) and melon‐headed whales (SPLASH, *n* = 5; SPOT, *n* = 3) within 12 km of Palmyra Atoll. We attached tags to the dorsal fin in the LIPMET configuration using two 45‐mm darts with three backward‐facing petals (Andrews et al. 2008, 2019) and we deployed the tags using a DAN‐INJECT JM 25 pneumatic projector rifle (DanWild LLC, Austin, TX, USA). SPOT tags recorded locations while SPLASH tags recorded locations and depth. SPLASH10‐F tags also recorded GPS locations (*n* = 2 melon‐headed whales and *n* = 2 bottlenose dolphins). All tags had a 30 s repetition rate. We collected video during each tag deployment using a Contour camera (Seattle, WA, USA) in wide angle. We collected photo‐identification images opportunistically using camera models Canon EOS 7D and Canon EOS R6 outfitted with a telephoto lens.

#### 2.1.3 Fishes

We caught yellowfin tuna (*n* = 15) and blue marlin (*n* = 1) within 40 km of Palmyra Atoll on artificial lures trolled on rod and reel fishing equipment and secured them in handling cradles or slings with their gills irrigated with running seawater during the tagging process. Reef manta rays (*n* = 8) were tagged within 9 km of Palmyra Atoll by a free diver with a hand‐powered tagging applicator (Hawaiian sling). We caught grey reef sharks (*n* = 8) within 12 km of Palmyra Atoll via baited handlines with barbless circle hooks and brought them alongside the boat for tagging. We attached pop‐up satellite archival tags (Wildlife Computers MiniPAT‐348) by inserting an applicator tip with a titanium dart at the tip into the epaxial muscle at the base of the dorsal fin on grey reef sharks, the second dorsal fin on yellowfin tuna, the first dorsal fin of the blue marlin, and to the posterior dorsal muscle on reef manta rays. All tags were attached to the dart via a custom‐built tether (Wilson et al. 2015). We measured total body length for grey reef sharks and caudal fin length and fork length for yellowfin tuna, the blue marlin, and grey reef sharks to the nearest cm with a measuring tape.

#### 2.1.4 Permits

Field work at Palmyra Atoll was conducted with the following permits: U.S. Fish and Wildlife Service Special Use Permits #12533–22,003, #12533–22,011, and #12533–22,012; U.S. Geological Survey (USGS) Bird Banding Lab permits #23411 and #23843; and U.S. National Marine Fisheries Service permit #20311. Field work was approved by the Institutional Animal Care and Use Committee (IACUC) at San Jose State University (#1077), Stanford University (#10765 and #10786), University of Washington (#4525–02), and USGS Animal Care and Use Committee (#WERC‐2007‐03).

### 2.2 Tracking Data Processing

We decoded tracking data with software from respective tag manufacturers (i‐gotU: @trip‐PC, version V5.0.1601.472; Lotek Pinpoint Host, version AV 2.15.1.0; Lotek Argos‐GPS Processor, version 4.2; Technosmart Axy‐Trek X‐Manager, version 1.16.4.22402; Wildlife Computers Data Processing Center, version 3.0.625) or with freely available online tools (e‐obs: www.movebank.org, version 14).

For fish tags, the most probable locations from MiniPAT geolocation tags were generated by the Wildlife Computers Global Position Estimator 3 (GPE3) statistical processing tool (Pedersen et al. 2011). The GPE3 model used tag‐derived light levels, temperature, and depth observations, as well as remotely sensed sea surface temperature (SST; NOAA OS SST V2 High Resolution; http://www.esrl.noaa.gov.psd/) and bathymetry (ETOPO1; Amante and Eakins 2009) in a hidden Markov model. GPE3 computes the joint posterior probability distribution of location and behavior on a 0.25° × 0.25° spatial grid throughout continuous time. Results were then interpolated to a smaller grid (0.025° latitude and longitude) and smoothed with a cubic spline within the GPE3. We conducted multiple GPE3 model runs per tag with incrementally increased species‐specific speed thresholds (Andrzejaczek et al. 2021; Curnick, Andrzejaczek, et al. 2020; Filous et al. 2022) and we selected the model with the highest quality score per individual for use in subsequent analyses (Table S1; e.g., Andrzejaczek et al. 2021). Geolocation tags sometimes had data gaps; we used interpolated data (from the GPE3) for data gaps < 20 days in duration and did not interpolate gaps > 20 days, and segments surrounding gaps were considered to be the same track for a given individual.

For cetacean tags, we uploaded raw Argos data from SPLASH and SPOT tags to Movebank (www.movebank.org) and we applied a Douglas‐Argos distance, angle, and rate (DAR) filter (Douglas et al. 2012) to the Argos Doppler locations (Hill et al. 2019). Analysis parameters followed Baird et al. (2021). We then added SPLASH‐Fastloc tags that also recorded GPS positions to the Douglas‐Argos filtered points, and we used these combined points in subsequent analyses. Pressure transducers provided depth estimates for three SPLASH tags.

We conducted all other tracking data processing and analyses in the program R (version 4.3.0; R Core Team 2023). We trimmed each animal's track to occur between its deployment start and end dates. We determined deployment end dates of tracks recorded by geolocation tags by visually inspecting pressure‐depth‐temperature time‐series plots for sudden departures in temperature and/or depth trends associated with normal diving behaviors. Deployment end dates for all other tags occurred when the animal was recaptured and the tag was removed (seabirds) or when the tag stopped transmitting data (seabirds and cetaceans).

For seabird locations, we applied species‐specific speed limits to all points (red‐footed booby: 40 m s^−1^, Adams et al. 2020; sooty tern: 14 m s^−1^, Soanes et al. 2015; great frigatebird: 25 m s^−1^, Weimerskirch and Prudor 2019) because this was not part of the tag processing steps, unlike the cetacean and fish tags. Because we tracked nesting seabirds that regularly make central place foraging trips from their nest but also spend many hours at or near their nest, we removed all nest‐centered points that occurred within 5 km of Palmyra Atoll. Then, for birds tracked for multiple days, we delineated each central place foraging trip with the function “MakeTrip” from the R‐package “trakR” (version 0.0.11; Fleishman et al. 2022) when trips traveled > 5 km from Palmyra and lasted at least 1 h (red‐footed booby and great frigatebird) or 6 h (sooty tern).

To standardize tracking data across tag types and species, we applied correlated random walks (CRW) to seabird and cetacean tracks with the function “fit_ssm” (R‐package “foieGras,” version 0.7–6; Jonsen and Patterson 2020), which generated points that were evenly spaced in time; we used vmax thresholds set to 5.6 m s^−1^ for bottlenose dolphins and melon‐headed whales, 25 m s^−1^ for great frigatebirds, 41.7 m s^−1^ for red‐footed boobies, and 13.9 m s^−1^ for sooty terns. The temporal resolutions of the resulting interpolated tracks matched tag sample rates and were 5 min (great frigatebird and red‐footed booby), 6 h (sooty tern), and 12 h (bottlenose dolphin and melon‐headed whale). We did not apply additional interpolation to the fish tracks because the GPE3 model had already estimated geolocations for 12 h time steps. To enable interspecific comparisons in habitat use, we estimated daily geodesic median locations per individual using the R‐package “Riemann” (version 0.1.4; You 2022), which we then used to estimate kernel densities and for some species distribution models (SDMs; see next section). Because many species exhibit distinct diurnal and nocturnal behaviors, each “day” corresponded to a 24 h period in the local time zone (HST) for this calculation.

To assess species movements (daily locations) within and outside PKMPA and U.S. EEZ boundaries, we estimated the overlap between each track and PKMPA and EEZ polygons with the R package “sf” (version 1.0–5; Pebesma 2018). Polygons of the EEZ were downloaded from www.marineregions.org.

### 2.3 Habitat Use

#### 2.3.1 Present Conditions

We used kernel density estimates to quantify species space use within and outside PKMPA with 50% and 95% utilization distributions (UD). We estimated UDs separately for daily median locations that occurred either inside or outside PKMPA boundaries. UDs calculated for distinct inside‐PKMPA/outside‐PKMPA regions enabled us to quantify and understand where each species was likely to occur under the current PKMPA configuration and could additionally inform future conservation efforts outside PKMPA. To do this, we first calculated whether each daily median location occurred inside or outside PKMPA with the function “st_contains” from the R package “sf” (Pebesma 2018). Then, we made separate estimates per species with the function “kernelUD” from the R package “adehabitatHR” (version 0.4.19; Calenge 2006), with a bivariate normal kernel and an h‐value of “href” over 150 grid intervals, with a cell size (extent) of 1° for all species (Young et al. 2015). We calculated the areas of estimated habitats inside and outside the MPA with the function “kernel.area” (Calenge 2006).

To understand how subsurface diving species used bathymetric depth in relation to maximum dive depth and PKMPA boundaries, we used linear mixed models with individual as a random effect with the function “lmer” from the R‐package “lme4” (version 1.1–29; Bates et al. 2015). We then used type 3 ANOVAs to assess the effects of species, MPA boundary, and bathymetric depth on dive depth.

We used species distribution models to characterize habitat use for each species except blue marlin, because we only tracked one individual. To do this, we used daily median locations in combination with pseudo‐absences to assess habitats that were both used and not used by animals. We generated pseudo‐absences following Hazen et al. (2021). Briefly, we simulated two types of pseudo‐absences: background and CRW. The extent over which pseudo‐absences were sampled was the maximum extent (minimum–maximum latitudinal and longitudinal ranges) of tracks per individual. Background sampling generated pseudo‐absence points within each individual's maximum spatial extent, whereas the pseudo‐absence points generated by CRW were constrained by the distributions of inter‐point distances and inter‐point angles between points in the original dataset. Daily median locations were used to generate pseudo‐absences with a 1:1 ratio of presences to pseudo‐absences. However, some individuals were only tracked for 1–3 days, resulting in few (1–3) locations per individual, which were insufficient to conduct simulated CRW. Therefore, we conducted simulated CRW twice, using two temporal resolutions: daily (simulated CRW was calculated using the geodesic median location—see previous section; *n* = 45 individuals) and the original resolution (simulated CRW was calculated using the original temporal resolution of each respective tag, which ranged 2 min–12 h, and then the geodesic median location was calculated from simulated CRW outputs; *n* = 75 individuals). We generated one hundred simulations of background sampling and 100 simulated correlated random walks per individual, per temporal resolution, and then we generated a presence‐absence track for each individual by randomly sampling from the 100 simulations.

We appended remotely sensed environmental covariates to each individual's presence‐absence track. We downloaded environmental variables from E.U. Copernicus Marine Service Information with a monthly resolution; dataset‐specific references are cited below. All remotely sensed datasets were level‐4 reprocessed products, which incorporate historical and near‐real time satellite and in situ observations into a reprocessed product that fills missing data values with temporal averaging or interpolation. This approach is useful for remote tropical ocean regions that are frequently covered by clouds. SST data (data title: “METOFFICE‐GLO‐SST‐L4‐NRT‐OBS‐SST‐V2”, DOI: https://doi.org/10.48670/moi‐00165) were from the global ocean OSTIA sea surface temperature and sea ice analysis dataset with a spatial resolution of 0.05° latitude and longitude (5.6 km) and used data from 1985 to present (Donlon et al. 2012; Good et al. 2020; Stark et al. 2007). Chlorophyll‐*a* data (data title: “cmems_obs‐oc_glo_bgc‐plankton_my_l4‐gapfree‐multi‐4km_P1D”, DOI: https://doi.org/10.48670/moi‐00281) were from global ocean color bio‐geo‐chemical L4 satellite observations with a spatial resolution of 0.04° (4.4 km) and used data from 1997 to present (E.U. Copernicus Marine Service Information (CMEMS) 2025b). Dissolved surface oxygen (DO) concentrations (data title: “global‐analysis‐forecast‐bio‐001‐028‐daily”, DOI: https://doi.org/10.48670/moi‐00015) were from the global ocean biogeochemistry analysis and forecast with a spatial resolution of 0.25° (27.8 km) and used data from 2008 to present (E.U. Copernicus Marine Service Information (CMEMS) 2025a). The u and v components of surface currents (data title: “cmems_mod_glo_phy‐cur_anfc_0.083deg_P1D‐m”, DOI: https://doi.org/10.48670/moi‐00016) were from the global ocean physics analysis and forecast with a spatial resolution of 0.083° (9.2 km) and used data from 2016 to present (E.U. Copernicus Marine Service Information (CMEMS) 2025c). We calculated surface current velocity with the equation $\sqrt{(u^{2}}+v^{2})$ and surface current heading with the function “atan2” (R Core Team 2023). Bathymetric depth data were obtained via the function “get.NOAA.bathy” from the R‐package “marmap” (version 1.0.6; Pante and Simon‐Bouhet 2013) from NOAA National Centers for Environmental Information ETOPO1 database (Amante and Eakins 2009) with a spatial resolution of 0.0166° (1.8 km) and used data from 1999 to 2008. We centered and scaled environmental variables prior to statistical analyses.

We then used presence‐absence locations in three statistical approaches to predict species occurrence at sea: boosted regression trees (BRT), generalized linear mixed models (GLMM), and generalized additive mixed models (GAMM). We selected these three approaches to generate a broader picture of model explanatory power within the context of ecological realism (e.g., Braun et al. 2023). Species‐specific binomial models predicted the probability of presences or absences based on environmental variables. We fit BRTs with the function “gbm.fixed” from the R‐package “dismo” (version 1.3–5; Hijmans et al. 2021) using the parameters learning rate = 0.005, tree complexity = 5, trees = 2000, and bag fraction = 0.75. We fit GLMM and GAMM with the function “gam” from the R‐package “mgcv” (version 1.8–38; Wood 2011), using individual animal as a random effect; we fit GAMMs with thin‐plate spline smoothers with 5 knots. We then assessed the performance (model explanatory power and predictive skill) for each statistical approach with three metrics: R^2^ and 10‐fold cross‐validation that generated area under the receiver characteristic curve (AUC) and true test statistics (TSS) following Hazen et al. (2021). Additionally, we used a measure of statistical independence, Bhattacharyya's coefficient, to assess environmental dissimilarity between presences and pseudo‐absences. Preliminary results indicated that background sampling with BRT consistently returned the best predictive skill and model fits (Table S2; Supplemental Text S1). Therefore, we used the models from the background sample‐BRT approach in SDMs to predict each species probability of occurrence at‐sea, which we interpret to indicate habitat suitability, over a larger area (a sample grid with size equal to the maximum extent of all species tracks) for two dates: June 01, 2022 (representing boreal spring/summer conditions) and December 01, 2022 (representing early boreal winter conditions) with the function “predict.gbm” from the R‐package “gbm” (version 2.1.8.1; Greenwell et al. 2022). We calculated median habitat suitability per species for PKMPA and the U.S. EEZ and plotted rasters of habitat suitability to visualize model results. To provide context for habitat suitability results, we chose the threshold of ≥ 0.67 to be interpreted as “highly suitable habitat.” In addition to 10‐fold cross validation, we evaluated SDMs with spatial block cross validation to account for autocorrelation, which partitioned the sample grid into blocks and assigned all observations in each block to either a training or test fold; this was repeated for five folds with the R‐package “blockCV” (version 3.1–1; Valavi et al. 2019). We visually determined species‐specific block sizes that balanced the number of observations and each species' spatial range with the function “cv_block_size” (block sizes: reef manta ray, yellowfin tuna: 500 km^2^; grey reef, shark, sooty, tern, great frigatebird: 200 km^2^; red‐footed booby: 76 km^2^; melon‐headed whale, bottlenose dolphin: 50 km^2^). Mean AUC values from 10‐fold cross validation and spatial block cross validation were assessed for overfitting by examining the difference between cross validation AUC with AUC from the original BRT models (Table S3); low difference values (e.g., < 0.1) were interpreted as performing well (e.g., Dedman et al. 2017).

#### 2.3.2 Climate Change Scenarios

To assess how species distributions might change under varying climate scenarios, we used the same modeling and evaluation approach as above to construct SDMs with environmental variables from the Climate Model Intercomparison Project Phase 6 (CMIP6) dataset (Eyring et al. 2016). We downloaded monthly datasets for SST, chlorophyll‐*a*, dissolved surface oxygen concentrations, and the u‐ and v‐components of surface current vectors with Python (version 3.11; Python Software Foundation, https://www.python.org/) via the Pangeo Gallery hosted by Google Cloud (https://gallery.pangeo.io/repos/pangeo‐gallery/cmip6/index.html). We obtained all available datasets for the “historical” time period and for two climate scenarios (SSP 1–2.6, “sustainability” and SSP 3–7.0, “rocky road”; Table S4). We chose these two climate scenarios because they provided a low and a high probabilistic projection and avoided the more extreme results predicted by other scenarios (e.g., SSP 5–8.5; Hausfather et al. 2022; Raftery et al. 2017). For each variable, we calculated the median value per month per grid cell separately across all historical and all future scenario datasets. We then subset historical and future scenario datasets for three time periods of interest by calculating a median value per month per grid cell for the historical reference period (1984–2014) and two future decades of interest: 2040–2050 and 2090–2100 for each of the two climate scenarios. We constructed SDMs using environmental variables from the historical reference period (not the environmental covariates concurrent with our tracking data, as used in the previous paragraph) because CMIP6 predictions were trained on models of the historical reference period (e.g., Archibald et al. 2024; Ling et al. 2024). We then applied this SDM to the set of variables from each of the two future decades of interest. Bathymetric depth (see above) was not expected to change under climate scenarios, but we added it to the sample grid to maintain consistency in the environmental covariates used among SDMs that represented current and future conditions. We centered and scaled all environmental variables prior to analyses. We subtracted results from the historical SDM from results from the future SDMs to quantify and visualize how species habitat suitability could change over time. Climate change analyses are not shown for bottlenose dolphins and melon‐headed whales because the CMIP6 environmental covariates were too spatially coarse (spatial resolution: 1° latitude and longitude) compared with the small extent of these species' tracks (< 100 km^2^). This resulted in little to no variation in most environmental variables, and thus models were not informative.

## 3 Results

**FIGURE 1:**
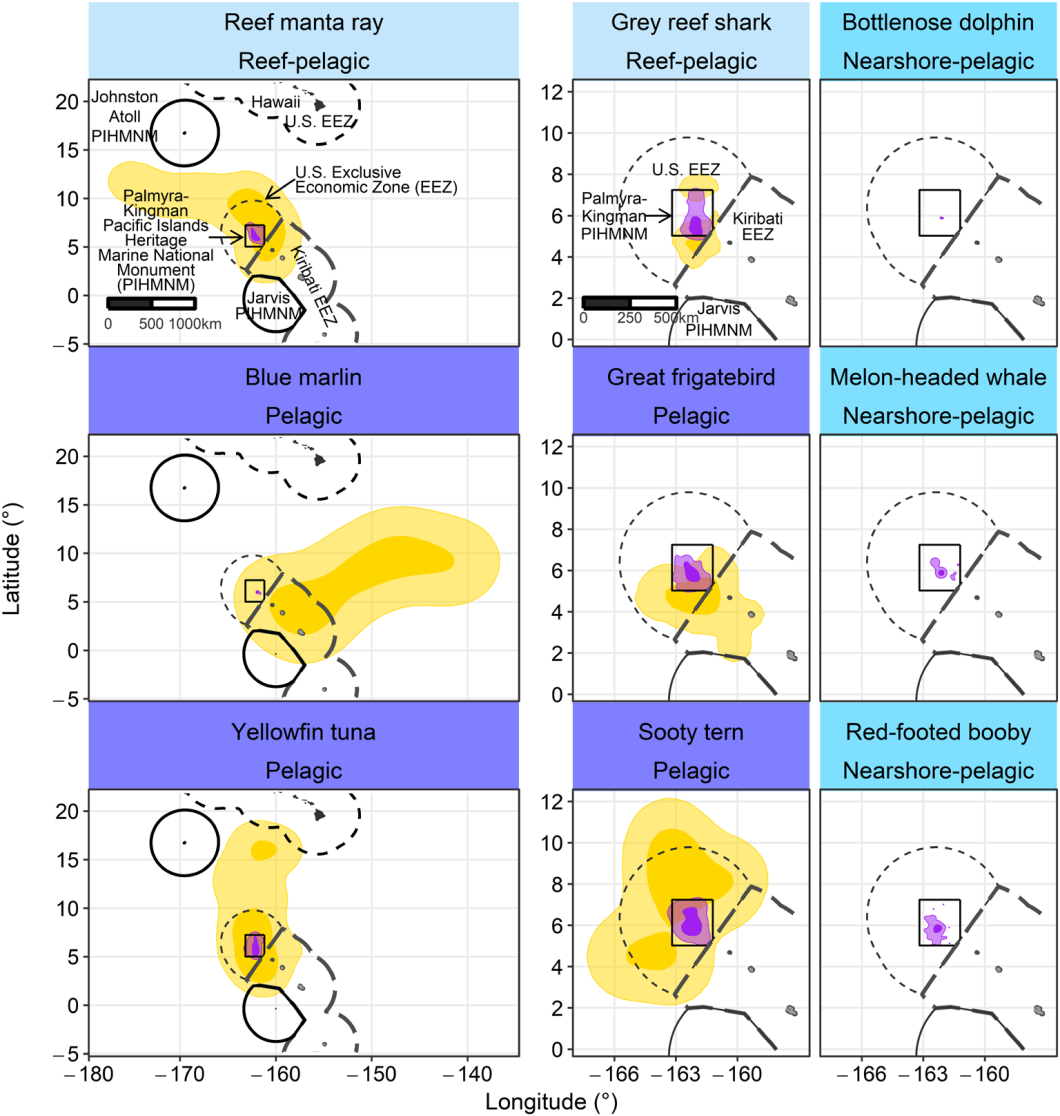
Species core and general use areas within and outside the Palmyra‐Kingman unit of the Pacific Islands Heritage Marine National Monument (PIHMNM) marine protected area (PKMPA). Utilization distributions were calculated separately for points inside (purple polygons) and outside (yellow polygons) PKMPA (solid black rectangle) during 2022–2023; due to the smoothing factor, polygons may appear to overlap PKMPA boundaries. Dark and light shading represent the 50% and 95% isopleths of kernel estimates, respectively. Dashed black lines indicate the U.S. Exclusive Economic Zone (EEZ) around PKMPA and Hawaiʻi. To provide regional geographic context, the Johnston Atoll and Jarvis Island units of PIHMNM (solid black polygons) and the Kiribati EEZ (dotted black line) are also shown. Map lines delineate study areas and do not necessarily depict accepted national boundaries.

Eighty‐three animals were tracked for 0.5–350 days (mean ± SD tracking duration: 39.8 ± 77.7 days; Table 1). Tracking durations varied between species due to tag types, premature tag detachment, predation, and technical battery issues. Bottlenose dolphins remained closest to Palmyra Atoll (< 50 km) and the blue marlin traveled farthest (> 2400 km). To aid in the interpretation of results, species were classified *post hoc* into three habitat groups based on mean maximum distances traveled from Palmyra Atoll and the locations of the core area (50% UD): nearshore–pelagic (mean: 23.3 km; max: 65 km; core area within 50 km of Palmyra Atoll; bottlenose dolphin, melon‐headed whale, red‐footed booby), reef–pelagic (mean: 415.5 km; max: 1679; core area within 500 km of Palmyra; reef manta ray, grey reef shark), and pelagic (mean: 245.7 km, max: 1256 km, and core area > 500 km from Palmyra; yellowfin tuna, sooty tern, great frigatebird; Figure 1).

### 3.1 How Does the MPA Protect Species Movements?

**FIGURE 2:**
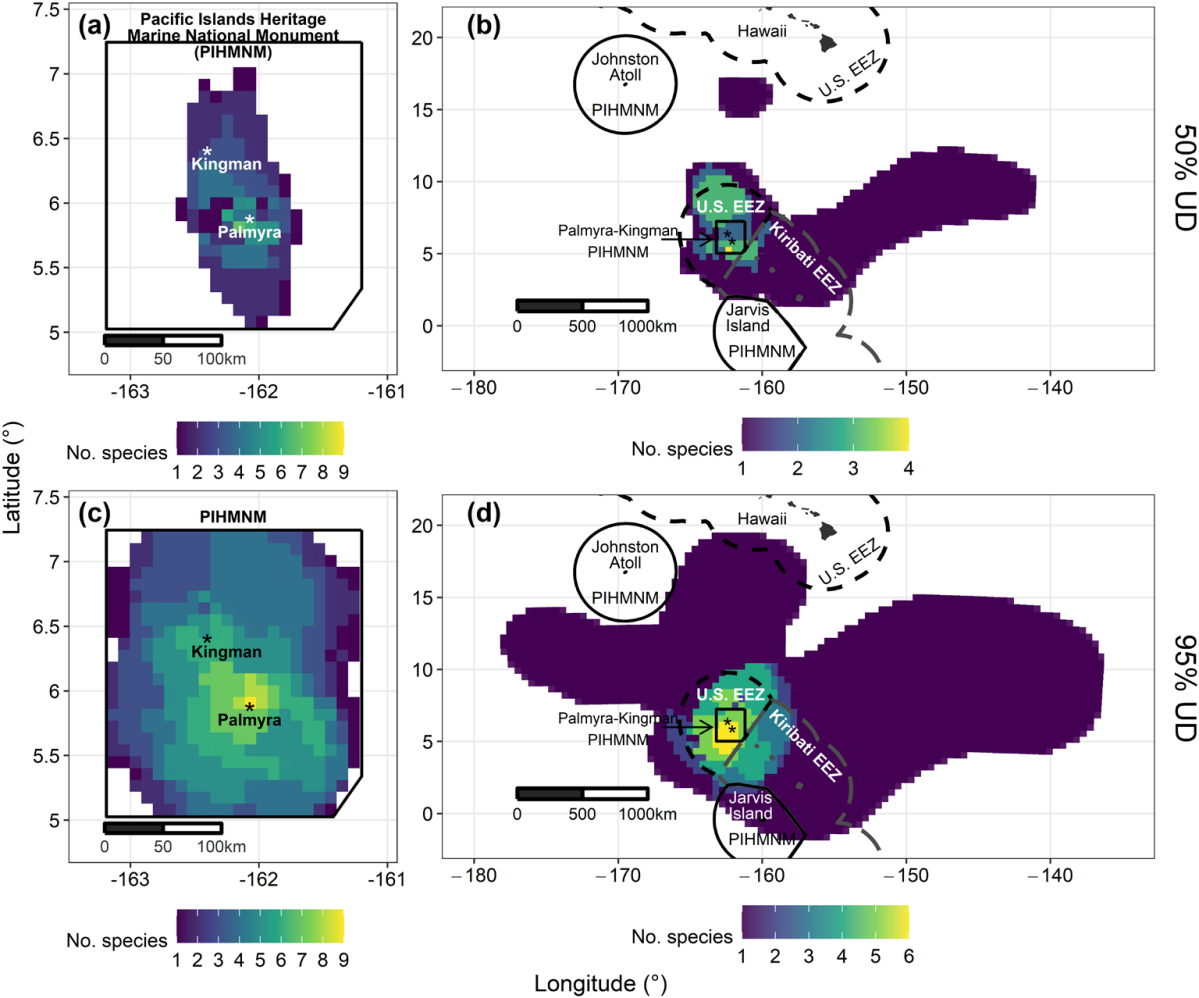
Combined species core and general use areas within (**a** and **c**) and outside (**b** and **d**) the Palmyra‐Kingman unit of the Pacific Islands Heritage Marine National Monument (PIHMNM) marine protected area (PKMPA; solid black line) in 2022–2023. The number of species with 50% utilization distributions (UDs; “core” area; **a** and **b**) and 95% UDs (“general” area; **c** and **d**) were summed per grid cell of size 10 km x 10 km (**a** and **c**) and 50 km x 50 km (**b** and **d**). The 10 km × 10 km grid cell size corresponds to a management relevant scale: A practicable area that could be monitored via small boat. To examine areas specifically within PKMPA, UDs were calculated separately for all points within and outside PKMPA. Because some species did not occur outside PKMPA, they were not included in the outside‐MPA calculations, resulting in fewer numbers of overlapping species (**b** and **d**). Feature labels are the same as Figure 1. Map lines delineate study areas and do not necessarily depict accepted national boundaries.

PKMPA and the U.S. EEZ encompassed 39% ± 46.4% and 54.4% ± 45.5%, respectively, of species movements (Table 1). Across all species, PKMPA contained a total of 4681 km^2^ of core‐use area, while 191,981 km^2^ of core‐use area occurred outside PKMPA. The highest concentration of species core (50% UD) and general‐use (95% UD) areas occurred within 50 km south, west, and northwest of Palmyra Atoll (Figure 2). Outside PKMPA, core and general‐use areas were concentrated southwest of PKMPA and north of the U.S. EEZ. Only three species (reef manta ray, sooty tern, great frigatebird) had core areas near Kingman Reef (Figure 1).

Species habitat groupings had varying overlap with PKMPA. Given their short dispersion distances and the durations of tag attachment, nearshore‐pelagic species (cetaceans, red‐footed boobies) occurred completely within PKMPA. Conversely, only 2.4% of reef manta ray and 29.3% of grey reef shark movements were within PKMPA. Two reef manta rays and all grey reef sharks made return trips to Palmyra Atoll, indicating reliance on both reef and pelagic regions. One reef manta ray traveled 1679 km northwest before its tag stopped transmitting (duration: 88 days). Wide‐ranging pelagic species (yellowfin tuna, sooty tern, great frigatebird) also used less area within PKMPA (5.2% ± 4.8%), and core‐use areas extended southeast into unprotected Kiribati waters and north and east into the unprotected High Seas. However, some general‐use areas extended into the Jarvis Island unit of PIHMNM (Figure 1).

Water column use by fishes and cetaceans ranged from the surface to > 1100 m deep (Table 1). Yellowfin tuna and cetaceans consistently dove to deeper depths than grey reef sharks and reef manta rays (Figure S1). One reef manta ray dove to 768 m, and one grey reef shark dove to 440 m, exceeding the deepest dives reported for these species (shark: 276 m Friedlander et al. 2017; manta: 672 m Lassauce et al. 2020). Maximum dive depths were not significantly different inside or outside PKMPA (type‐3 ANOVA: MPA boundary: *χ* ^2^ = 1.2, df = 1, *p* = 0.266; species: *χ* ^2^ = 45.9, df = 3, *p* < 0.0001). However, fishes dove significantly deeper when in deeper water (type‐3 ANOVA: bathymetric depth: *χ* ^2^ = 4.4, df = 1, *p* = 0.036; species: *χ* ^2^ = 41.2, df = 3, *p* < 0.0001).

### 3.2 How Does the MPA Protect Habitats?

#### 3.2.1 Habitat Requirements of Species

Suitable habitats were mainly defined by shallow to moderate bathymetric depths for all species except sooty terns (Table S5). Sooty tern habitat was equally characterized by all environmental variables, resulting in habitat with narrow ranges of moderate bathymetric depth, moderate chlorophyll‐*a* and dissolved O_2_ (DO) concentrations, and slow northeastward surface currents (Figure S2).

The remaining environmental variables had a mixed influence in the models. In addition to bathymetric depth, pelagic species habitats were described by moderate chlorophyll‐*a* concentrations and warm SST (relative model contributions of 11%–18%; Table S5; Figure S2). More broadly, surface currents and DO were important across nearshore, reef, and pelagic habitats. For example, like pelagic sooty terns, red‐footed booby (nearshore‐pelagic) habitat was characterized by low eastward surface current velocity; eastward and southward surface currents also contributed to great frigatebird (pelagic) habitat, and northward currents contributed to reef manta ray (reef‐pelagic) habitats. Low DO concentrations characterized yellowfin tuna (pelagic) and reef manta ray (reef‐pelagic) habitats.

#### 3.2.2 Occurrence of Habitats Within PKMPA, the U.S. EEZ, and the High Seas

**FIGURE 3:**
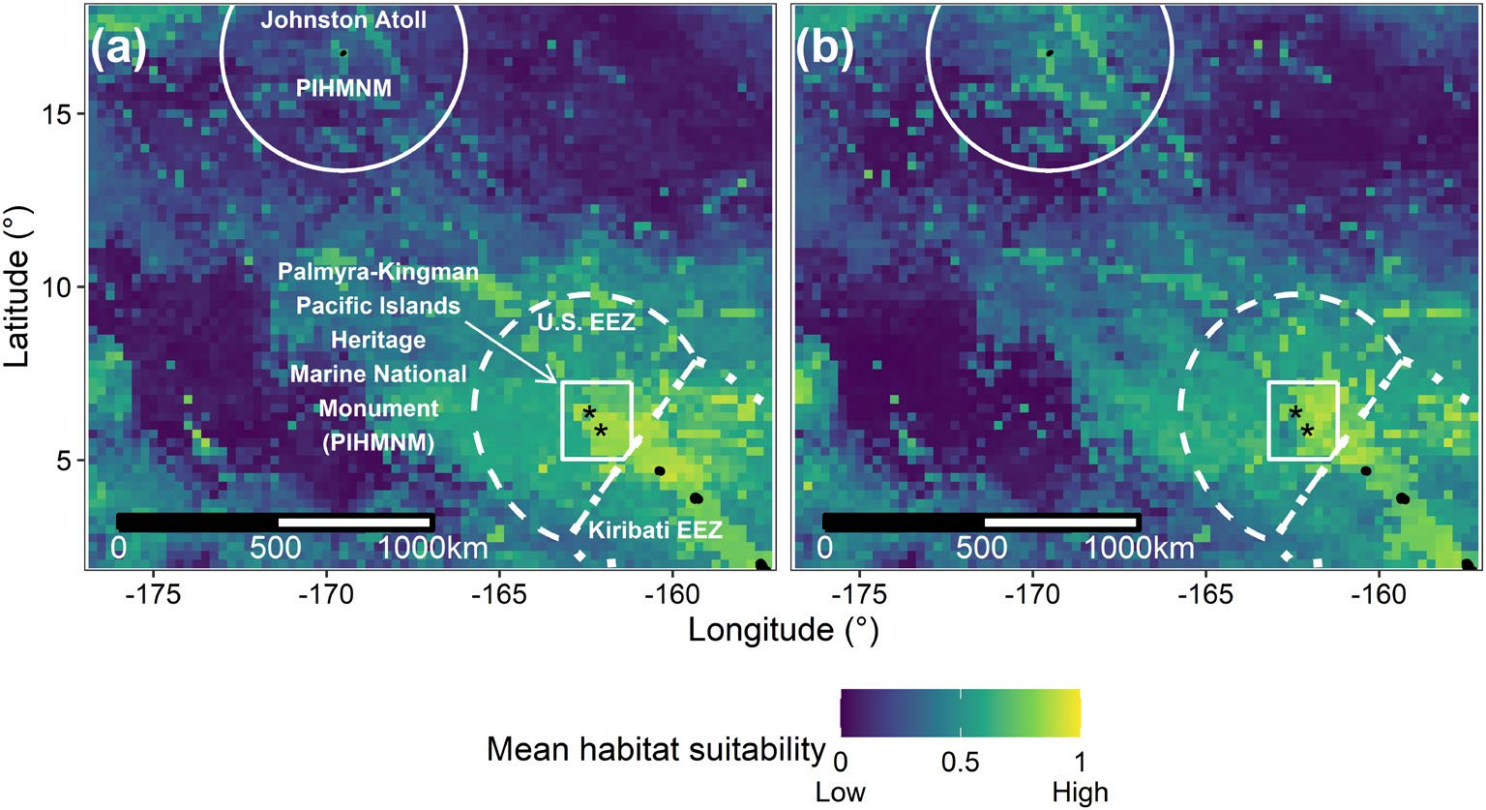
Mean habitat suitability within and outside the Palmyra –Kingman unit of the Pacific Islands Heritage Marine National Monument (PIHMNM) for (**a**) boreal spring/summer (June 1, 2022) and (**b**) early winter (December 1, 2022). Mean habitat suitability was calculated from eight species per 0.25 x 0.25° grid cell and is represented by a dark–light color scale, where dark colors indicate low habitat suitability and light colors indicate high habitat suitability. Feature labels are the same as Figure 1. Map lines delineate study areas and do not necessarily depict accepted national boundaries.

PKMPA contained highly suitable habitats (habitat suitability values > 0.67) and highly suitable habitat also occurred in Johnston Atoll MPA during early winter (Figure 3). Nearshore‐pelagic habitats were seasonally variable: bottlenose dolphins gained 25% in PKMPA habitat by early winter, while all other species lost habitat (−3% to −31%, though yellowfin tuna gained 4%; Figures S3, S4). In the larger EEZ, red‐footed boobies gained 83% habitat by winter, but melon‐headed whales lost 63% habitat.

**FIGURE 4:**
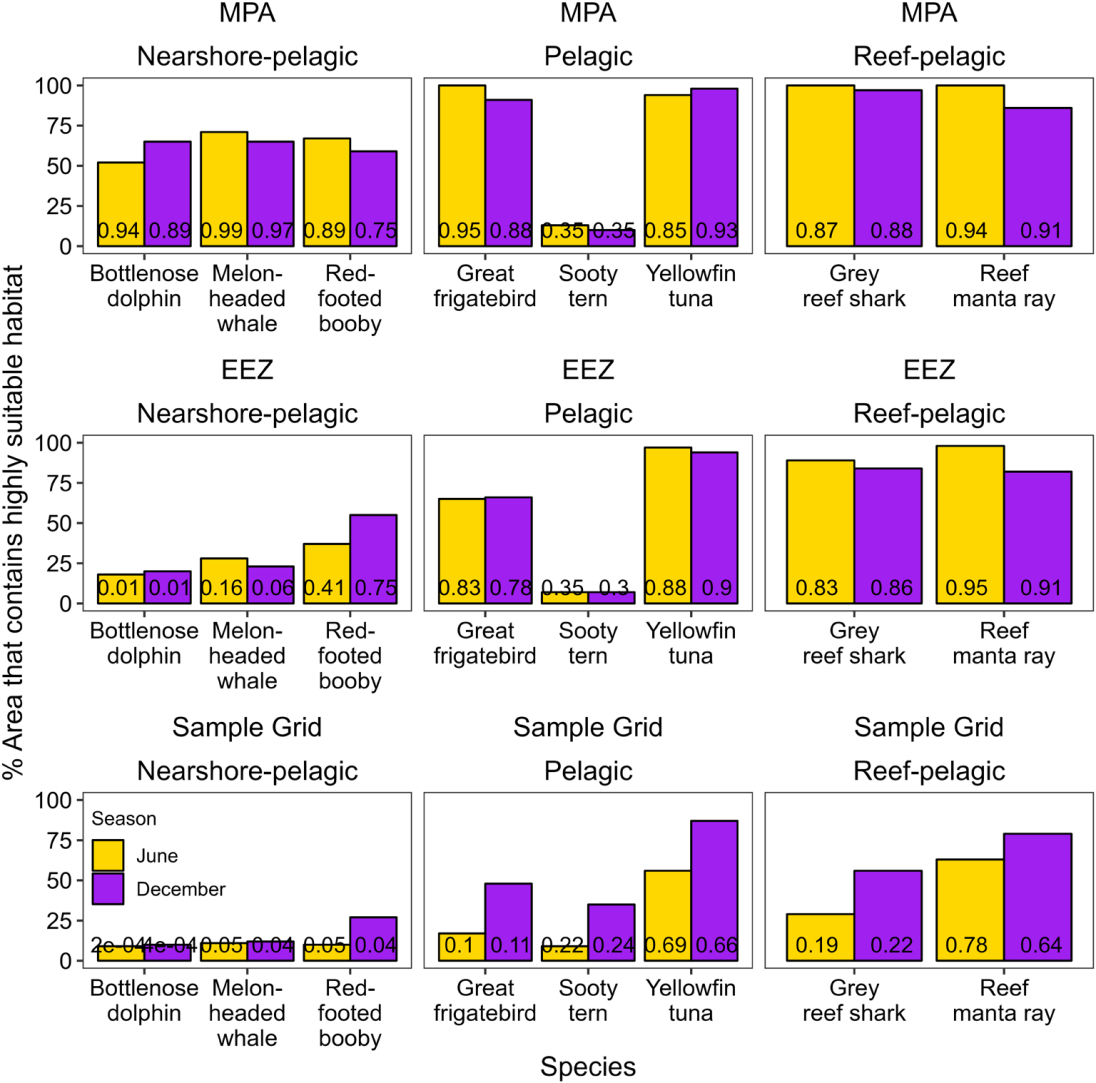
Available suitable habitat varied between nearshore‐pelagic, pelagic, and reef‐pelagic regions but changed little between seasons for most species. Bar plots represent the percent highly suitable habitat (> 0.67) within the Palmyra‐Kingman Marine Protected Area (MPA), the U.S. Exclusive Economic Zone (EEZ) surrounding PKMPA, and the whole sample grid (see Figure 3). Values on bars correspond to the median habitat suitability within PKMPA in boreal spring/summer (June; yellow) and early winter (December; purple).

Narrow ranges of environmental covariates important to sooty terns resulted in only 13% of PKMPA containing highly suitable sooty tern habitat, compared with 94%–100% for other pelagic and reef‐pelagic species (Figure 4). Similarly, only 52%–71% of PKMPA contained highly suitable habitat for nearshore‐pelagic species, even though they spent 100% of their time in PKMPA.

Highly suitable habitats extended into unprotected areas. Most species had highly suitable habitat within Kiribati's EEZ during both seasons, specifically surrounding three atolls (Teraina [Washington Island], Tabuaeran [Fanning Island], Kiritimati [Christmas Island]). Highly suitable habitat also extended into the High Seas (approximately 150 km east and west of PKMPA) for all species except nearshore bottlenose dolphins, melon‐headed whales, and pelagic sooty terns (**Figures** S3, S4).

### 3.3 How Might the MPA Protect Species Habitat as Climate Changes?

**FIGURE 5:**
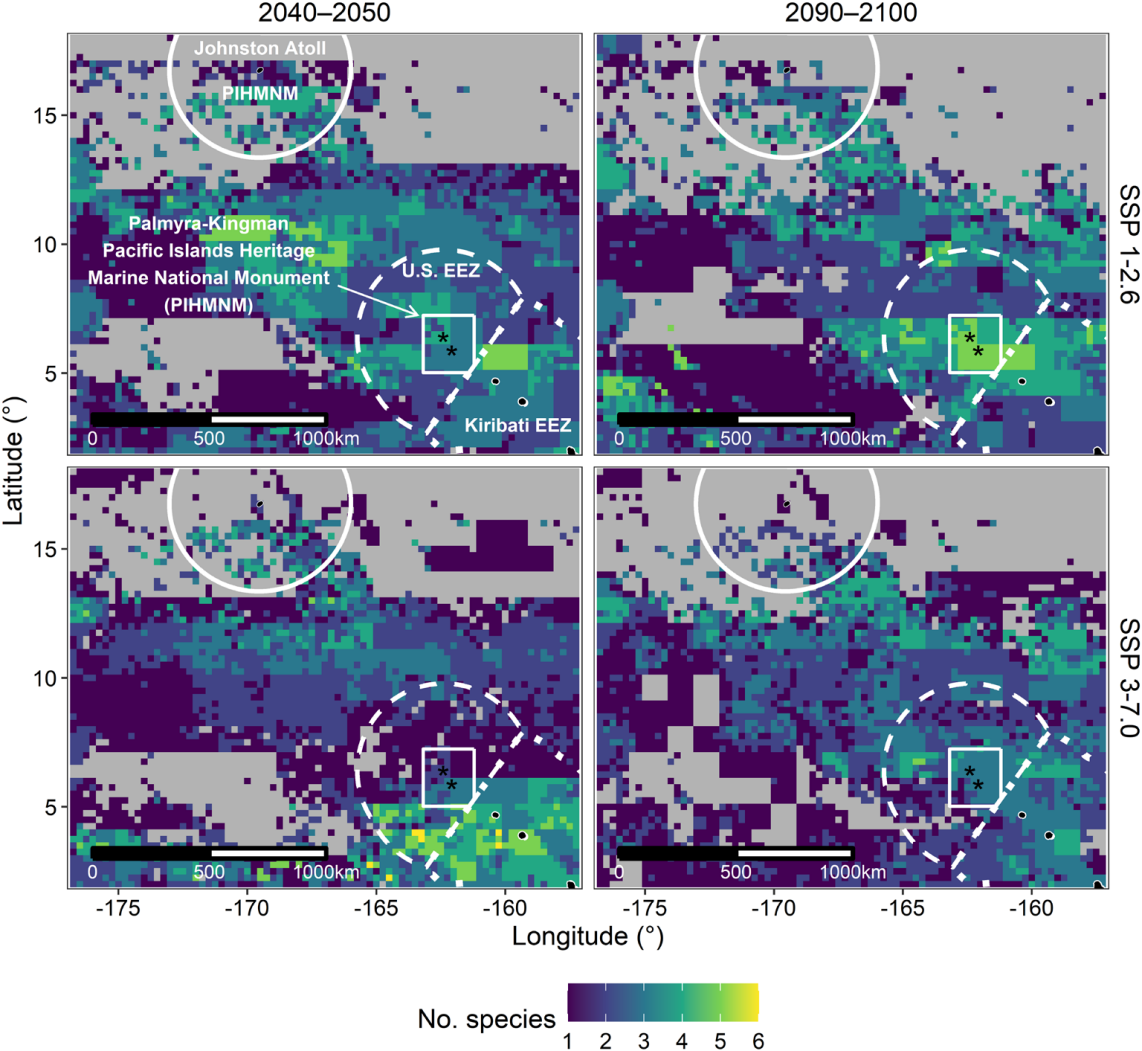
The sum of species with high habitat suitability (> 0.67) per 0.25°x 0.25°grid cell for each climate scenario (rows; SSP 1–2.6, SSP 3–7.0) and time period (columns). Grey‐colored cells indicate areas in which zero species had high habitat suitability. Feature labels are the same as Figure 1. Map lines delineate study areas and do not necessarily depict accepted national boundaries.

Highly suitable habitats occurred in 52% ± 44% of PKMPA under two climate scenarios and during the two time periods (2040–2050 and 2090–2100; Table S6; Figure 5). Highly suitable habitat in PKMPA decreased during 2040–2050 by 34% ± 32% under SSP 1–2.6 (“Sustainability”) and by 41% ± 28% under SSP3‐7.0 (“Rocky Road”). During 2090–2100, much smaller habitat changes were predicted in PKMPA under both scenarios (SSP 1–2.6: −5% ± 33%; SSP 3–7.0: −17% ± 26%) compared with 2040–2050. The greatest predicted mean change over time occurred for reef‐pelagic and nearshore‐pelagic species, resulting in substantial species‐specific habitat losses and gains in PKMPA and the EEZ (Figure S5). For example, red‐footed boobies lost > 50% habitat during 2040–2050 and grey reef shark habitat decreased by 82% during 2040–2050; however, grey reef shark habitat nearly tripled in 2090–2100. Conversely, pelagic species experienced relatively smaller changes except for great frigatebirds, which experienced the greatest mean habitat loss of all species in 2040–2050 (Table S7).

Outside PKMPA, most highly suitable habitats occurred to the south and east (within the Kiribati EEZ) and in the corridor region located northwest of PKMPA and the southern boundary of Johnston Atoll (covering approximately 250 km from northwest to southeast; Figure 5), such that most species gained habitat in this corridor under both time periods and climate scenarios (Figure S5).

## 4 Discussion

### 4.1 Does the MPA Protect Species Movements & Habitats?

Our large‐scale study took a unique approach to assess the efficacy of a LSMPA by concurrently tracking multiple, wide‐ranging species that used a common LSMPA and then integrating telemetry with remotely sensed environmental conditions and climate change scenarios to characterize current and future habitats. Concurrently tracking species enabled us to examine all species' responses to the same environmental conditions and estimate habitat suitability with the most up to date information. Our approach is especially timely within the context of ongoing environmental change and efforts to expand PKMPA (Biden 2023). Although we highlight areas of importance surrounding PKMPA, our results can also provide a framework for other MPA assessments and are globally relevant to many nations attempting to meet Target 3 of the Kunming‐Montreal Global Biodiversity Framework (protecting 30% of waters by 2030; “30 X 30”) by establishing LSMPAs.

#### 4.1.1 Protection of Species Movements

PKMPA contains abundant resources and diverse habitats that contribute to high biodiversity and accrue climate resilience benefits, but its current configuration encompasses < 50% of species movements, and 6/9 species traveled to unprotected areas. Species ecology and life history can, however, help quantify protections and risks at varying spatiotemporal scales. For example, although melon‐headed whales and bottlenose dolphins remained near Palmyra, their diet is not well understood for island populations (Young et al. 2017) and unknown changes in prey species ecology could affect travel within and outside PKMPA. Similarly, cross‐boundary foraging bouts by central‐place foragers like breeding seabirds occur at short time scales (hours to days) because they move directly to specific habitats or pursue mobile prey species (e.g., Madden et al. 2022). The brevity of cross‐boundary movements may result in overall greater protection. However, being near boundaries could be problematic because, although resources adjacent to MPAs are enhanced (Caselle et al. 2015), fisheries activity may also be higher, and regional enforcement efforts may be limited (Jacoby et al. 2020; White et al. 2017). Longer movements associated with migration and breeding (time scale: weeks to months) that were not captured in this analysis due to tagging limitations can occur during critical life history phases that require transit to, or away from, PKMPA. For example, spawning habitats have not been identified for tuna in the region (Hernández et al. 2019), resulting in unknown movements during reproduction. At a larger scale, global stressors like climate change can adversely affect animals within and outside MPAs, superseding local protective and enforcement efforts (Maxwell et al. 2013). Improved life history knowledge combined with more comprehensive habitat information can therefore better identify effective scales of MPA protection.

#### 4.1.2 Protection of Habitats

Well‐placed MPAs protect habitats within their boundaries, and many smaller, coastal MPAs are designed specifically to consider habitat (Blanluet et al. 2024). However, dynamic open ocean processes may only occur temporarily inside LSMPA boundaries (e.g., frontal zones provide foraging opportunities but are mobile and temporary Scales et al. 2014), providing a basis for species to remain within or leave PKMPA. Understanding relationships between topographic features and ephemeral processes is essential to fully characterize habitats and understand the extent to which they occur within an MPA over the annual cycle (Visalli et al. 2020). Bathymetric depth was the most important variable in nearly all SDMs, representing static habitat (including the island mass effect; Gove et al. 2016; and high seamount density; S.‐S. Kim and Wessel 2011) that can influence dynamic processes within PKMPA. Just outside PKMPA, two‐thirds of species regularly visited a region 100–150 km southwest of Palmyra. Grey reef sharks (White et al. 2017), red‐footed boobies (Young et al. 2015), and great frigatebirds (Gilmour et al. 2019) used this area in previous tracking studies. Here, enhanced oceanic mixing occurs between May–January from the physical shear between the equatorial current and counter‐currents (Maragos et al. 2008). Seasonal winds also enhance surface oxygen concentrations in this region, and O_2_ flux is greatest during boreal summer and fall (Eddebbar et al. 2024). Seasonal processes that result from such physical forcing likely enhance foraging opportunities for many predators. Important habitats may thus be better protected if associations between topographic and ephemeral features occur at predictable spatial or temporal scales (Gilmour et al. 2022).

The relative importance of ephemeral environmental features varied among species models, but some similarities illustrated potential interspecific connections. For example, because some seabirds rely on facilitated foraging to obtain subsurface prey (Maxwell and Morgan 2013), sooty terns and yellowfin tuna exploit similar habitats. This might explain the importance of DO in tuna and sooty tern models because it is physiologically limiting to fish and affects their distribution (Stramma et al. 2012). However, interspecific interactions may also expose animals, including seabirds (Gilman et al. 2016) and sharks (Shea et al. 2023), to additional threats like fisheries bycatch. This highlights the benefits of examining multiple species and interspecific interactions within the context of marine spatial planning.

LSMPAs are hard to design because of the presence and potential importance of stationary and ephemeral non‐stationary features. Yet, the protection of diverse habitats within a single MPA is key to protecting biodiversity and ocean health. It is recommended that new MPAs encompassing multiple habitats be designed to include at least 30% of each habitat type (Arafeh‐Dalmau et al. 2023). In addition to stationary and ephemeral features, PKMPA encompasses both an atoll (Palmyra) and a guyot (Kingman) that generate different nutrient resources (reef‐derived nutrients at Kingman from consistent topographical and current‐driven upwelling; Brainard et al. 2019; vs. nutrients derived from forests and seabirds at Palmyra; McCauley, DeSalles, et al. 2012). Although only 24% of animals tagged at Palmyra Atoll traveled to Kingman Reef, Kingman hosts large numbers of reef sharks, tuna, and mantas (Brainard et al. 2019; Friedlander et al. 2010). The uniqueness of PKMPA and the many species that occur within it or traverse the region surrounding it emphasize the benefits of additional studies that consider animal movement and habitat characteristics for marine spatial planning and assessment.

Future studies with longer tracking durations with more individuals would improve information for the three habitat types that species used in this study. Our assessment was based on tracking data from nine diverse species that were collected for different lengths of time and had varying ranges of position estimate error. Species dispersal away from PKMPA may not have been fully captured, and the spatial scale of habitat models may be smaller than these species maximum ranges in the central Pacific. Indeed, because dispersal away from the tagging location is often proportional to tagging duration, models may have provided an atoll‐centric perspective for species tagged for short time periods (< 30 days) and may not have captured inter‐seasonal changes. However, movements were within range of previously reported behaviors for focal species, including previous tracking efforts at Palmyra between 2006 and 2014 (summarized in Gilmour et al. 2022), and additional longer term data would help refine current and future habitat assessments (Carlisle et al. 2019).

#### 4.1.3 Protection as Climate Changes

Although climate change causes coral bleaching at Palmyra (Khen et al. 2022), large mobile species reveal variable responses to future climate scenarios, with nearshore and reef species exhibiting the largest overall predicted habitat changes. Varying climate change effects are predicted for the equatorial Pacific Ocean. For example, increased SST and rainfall are expected to increase water column stratification (Collins et al. 2010), which in turn will likely weaken equatorial currents and decrease upwelled nutrients (H. Kim et al. 2023). Subsequent changes in eddy activity (Beech et al. 2022) could reduce foraging opportunities and decrease the spatiotemporal predictability associated with these features (e.g., Welch et al. 2023). Increased temperature and decreased oxygen degrade coral reefs, providing less coral cover for larval settlement, increasing algal presence, decreasing biodiversity (Nelson and Altieri 2019), and decreasing productivity that creates echelon effects on local food webs (Millington et al. 2022). Grey reef sharks and reef manta rays derive energy from coral reef and lagoon systems (McCauley et al. 2014; McCauley, Young, et al. 2012) and could have a greater sensitivity to these nearshore changes.

Unexpectedly, SDMs predicted overall habitat loss in the near term (2040–2050), but habitat gain was predicted for the more distant future (2090–2100). In the equatorial Pacific, an initially strong SST gradient is predicted by the mid‐21st^st^ century, followed by a weakened gradient that results in an equilibrium response by 2100 (Heede et al. 2020). A strong SST gradient could produce large, abrupt oceanic and ecological changes and habitat loss, potentially causing adverse changes in prey habitat and subsequent prey availability in PKMPA. Although species with warm‐water distributions and wide temperature tolerance are expected to expand habitats (Tompkins et al. 2017), the rate at which prey species adapt might be limited. In response, some predators may switch to different prey species within the same habitats (Magurran et al. 2015). A more comprehensive understanding of diet flexibility could inform responses to abrupt and long‐term ecological changes. Long‐term habitat gains predicted in PKMPA could be related to a combination of generalist diets and complex environmental processes that are not driven solely by temperature anomalies (Welch et al. 2023). Some have questioned the point of conservation in the face of climate change (Olson and Lindsay 2009), but the availability of some persistent suitable habitat under future climate scenarios indicates that well‐designed current protections, especially those that consider future scenarios, are a good long‐term investment.

Ongoing regional and multinational communication, monitoring, and enforcement will likely benefit future habitat protections (White et al. 2017). Current and future highly suitable habitat occurred within the Kiribati EEZ and at Johnston Atoll. Considering both highlights the need for communication and coordination with nearby central Pacific neighbors and demonstrates that monitoring and enforcement regimes among disparate units of LSMPAs could be factored into marine spatial planning and fisheries management now and in the future to maximize benefits (Boerder et al. 2019). This is especially important considering predicted near‐term habitat loss. For example, Johnston Atoll is a stepping stone for larval reef fish dispersal (Kobayashi 2006), and potentially also valuable for other species vulnerable to climate change‐induced migration. Additional holistic benefits could be realized for wide‐ranging, cross‐boundary species if coupled with fisheries management (Boerder et al. 2019). PKMPA could also buffer future habitat changes by protecting part of species ranges, thereby alleviating the severity of some stressors that animals may encounter in areas that are not under management efforts (e.g., on the High Seas; also see Maxwell et al. 2013). Collectively, these efforts could yield positive outcomes that more accurately reflect the spatial ecology of species with cross‐boundary movements.

### 4.2 How Might the MPA Protect Species if Configured Differently?

Evaluations of MPA efficacy have demonstrated that there is no one‐size‐fits‐all approach for MPA design, especially when considering the diversity of species within them (Conners et al. 2022; Gilmour et al. 2022). MPAs range in size from 1 km^2^ to > 100,000 km,^2^ and boundaries may be stationary, mobile, or comprise an MPA network. PKMPA is a large stationary MPA within the huge 1.2 million km^2^ PIHMNM network. This network protects multiple habitat types and linkages between ecosystems, including islands and lagoons, forests and coral reefs, and coral reefs and pelagic oceans. A holistic approach that conserves these habitats could enhance MPA protections of biodiversity and nearshore and pelagic food webs. If PKMPA were larger or connected more directly to neighboring PIHMNM units via animal movement corridors, its protections could likely improve. Such an expansion would encompass a larger contiguous area better able to accommodate mobile, far‐ranging species (predators and prey) that rely on dynamic ocean processes (Boerder et al. 2019; Pendoley et al. 2014) and species‐specific residential and migratory habitats (Dunn et al. 2019). When considering additional areas, our telemetry data and habitat models revealed that the region south of Palmyra contained important habitat for multiple species. Other protections, such as limits on FADs drifting through the MPA, might increase protection for commercially important fishes (Tolotti et al. 2020). Additionally, strategically located High Seas MPAs could offer ecologically informed buffers outside of EEZs in sensitive or important areas or help promote connectivity among areas (Maxwell et al. 2020). These considerations are timely due to a recent directive to consider expanding the size of PKMPA (Biden 2023) and the potential ratification of the High Seas Treaty (United Nations 2023a, 2023b).

### 4.3 Conclusions

Telemetry data demonstrated that marine species overlapped with PKMPA and thus received some implicit protections. Species movements also highlighted areas of importance within and outside PKMPA. More importantly, suitable habitats occur now and will remain within both PKMPA and in contiguous adjacent marine areas in the future. These contiguous areas were especially important as the marine climate changes; MPA expansion and future conservation efforts among neighboring islands and along corridors connecting atolls may provide long‐term benefits for species and help preserve existing biodiversity. MPAs are only effective for conservation if they are used in conjunction with other regulatory and enforcement efforts that address threats like illegal, unreported, and unregulated (IUU) fishing, derelict fishing gear, pollution, and climate change (Abessa et al. 2018; Curnick, Feary, et al. 2020). Moreover, an MPA's efficacy depends on its monitoring and enforcement (Jacoby et al. 2020). Given MPA benefits to adjacent populations (Medoff et al. 2022), a concerted effort among neighboring regions is especially important for future conservation and sustainability, and animal telemetry data can help quantify the significance of these benefits (Boerder et al. 2019). Although the dynamic needs of mobile species and the constantly changing ocean in which they live may not be fully met by stationary MPAs, dynamic MPAs and MPA networks together with other conservation actions can provide space to mitigate stressors faced by vulnerable marine fauna (Benedetti‐Cecchi et al. 2024).

## Conflicts of Interest

The authors declare no conflicts of interest.

## Supporting information


**Data S1** Supporting Information.

## Data Availability

The data that support the findings of this study are openly available from the Data Observation Network for Earth (DataONE) at https://doi.org/10.24431/rw1k8ez. Environmental variables were obtained from the E.U. Copernicus Marine Service Information: sea surface temperature https://doi.org/10.48670/moi‐00165; chlorophyll‐a https://doi.org/10.48670/moi‐00281; dissolved oxygen https://doi.org/10.48670/moi‐00015; and surface currents https://doi.org/10.48670/moi‐00016. Bathymetry data were provided by the NOAA National Geophysical Data Center https://doi.org/10.7289/V5C8276M. Sea surface temperature, chlorophyll‐a, dissolved oxygen, and surface current datasets under future predicted CMIP6 scenarios were obtained from the Earth System Grid Federation at https://esgf‐data.dkrz.de/search/cmip6‐dkrz/. The data that support the findings of this study are openly available from the Data Observation Network for Earth (DataONE) at https://doi.org/10.24431/rw1k8ez. Environmental variables were obtained from the E.U. Copernicus Marine Service Information: sea surface temperature https://doi.org/10.48670/moi‐00165; chlorophyll‐a https://doi.org/10.48670/moi‐00281; dissolved oxygen https://doi.org/10.48670/moi‐00015; and surface currents https://doi.org/10.48670/moi‐00016. Bathymetry data were provided by the NOAA National Geophysical Data Center https://doi.org/10.7289/V5C8276M. Sea surface temperature, chlorophyll‐a, dissolved oxygen, and surface current datasets under future predicted CMIP6 scenarios were obtained from the Earth System Grid Federation at https://esgf‐data.dkrz.de/search/cmip6‐dkrz/.

## References

[gcb70138-bib-0001] Abessa, D. M. S. , H. C. Albuquerque , L. G. Morais , et al. 2018. “Pollution Status of Marine Protected Areas Worldwide and the Consequent Toxic Effects Are Unknown.” Environmental Pollution 243: 1450–1459. 10.1016/j.envpol.2018.09.129. 30292154

[gcb70138-bib-0002] Adams, J. , J. J. Felis , and M. F. Czapanskiy . 2020. “Habitat Affinities and At‐Sea Ranging Behaviors Among Main Hawaiian Island Seabirds: Breeding Seabird Telemetry, 2013‐2016. (US Department of the Interior, Bureau of Ocean Energy Management, Pacific OCS Region OCS Study BOEM 2020‐006).” https://www.sciencebase.gov/catalog/item/5b6cded4e4b0f5d578752dcf.

[gcb70138-bib-0003] Amante, C. , and B. W. Eakins . 2009. “ETOPO1 1 Arc‐Minute Global Relief Model: Procedures, Data Sources and Analysis (NOAA Technical Memorandum NOAA Technical Memorandum NESDIS NGDC‐24.” https://www.ngdc.noaa.gov/mgg/global/relief/ETOPO1/docs/ETOPO1.pdf.

[gcb70138-bib-0004] Amon, D. J. , J. Palacios‐Abrantes , J. C. Drazen , et al. 2023. “Climate Change to Drive Increasing Overlap Between Pacific Tuna Fisheries and Emerging Deep‐Sea Mining Industry.” NPJ Ocean Sustainability 2, no. 1: 9. 10.1038/s44183-023-00016-8.

[gcb70138-bib-0005] Andrews, R. D. , R. W. Baird , J. Calambokidis , et al. 2019. “Best Practice Guidelines for Cetacean Tagging.” IWC Journal of Cetacean Research and Management 20, no. 1: 27–66. 10.47536/jcrm.v20i1.237.

[gcb70138-bib-0006] Andrews, R. D. , R. L. Pitman , and L. T. Ballance . 2008. “Satellite Tracking Reveals Distinct Movement Patterns for Type B and Type C Killer Whales in the Southern Ross Sea, Antarctica.” Polar Biology 31, no. 12: 1461–1468. 10.1007/s00300-008-0487-z.

[gcb70138-bib-0007] Andrzejaczek, S. , R. J. Schallert , K. Forsberg , et al. 2021. “Reverse Diel Vertical Movements of Oceanic Manta Rays Off the Northern Coast of Peru and Implications for Conservation.” Ecological Solutions and Evidence 2: e12051. 10.1002/2688-8319.12051.

[gcb70138-bib-0008] Arafeh‐Dalmau, N. , A. Munguia‐Vega , F. Micheli , et al. 2023. “Integrating Climate Adaptation and Transboundary Management: Guidelines for Designing Climate‐Smart Marine Protected Areas.” One Earth 6, no. 11: 1523–1541. 10.1016/j.oneear.2023.10.002.

[gcb70138-bib-0009] Archibald, C. L. , D. M. Summers , E. M. Graham , and B. A. Bryan . 2024. “Habitat Suitability Maps for Australian Flora and Fauna Under CMIP6 Climate Scenarios.” GigaScience 13: giae002. 10.1093/gigascience/giae002. 38442145 PMC10939329

[gcb70138-bib-0010] Baird, R. W. , C. J. Cornforth , S. M. Jarvis , et al. 2021. “Odontocete Studies on the Pacific Missile Range Facility in February 2020: Satellite Tagging, Photo‐Identification, and Passive Acoustic Monitoring.”

[gcb70138-bib-0011] Bates, D. , M. Maechler , B. Bolker , and S. Walker . 2015. “Fitting Linear Mixed‐Effects Models Using lme4.” Journal of Statistical Software 67, no. 1: 1–48. 10.18637/jss.v067.i01.

[gcb70138-bib-0012] Beech, N. , T. Rackow , T. Semmler , S. Danilov , Q. Wang , and T. Jung . 2022. “Long‐Term Evolution of Ocean Eddy Activity in a Warming World.” Nature Climate Change 12, no. 10: 910–917. 10.1038/s41558-022-01478-3.

[gcb70138-bib-0013] Benedetti‐Cecchi, L. , A. E. Bates , G. Strona , et al. 2024. “Marine Protected Areas Promote Stability of Reef Fish Communities Under Climate Warming.” Nature Communications 15, no. 1: 1822. 10.1038/s41467-024-44976-y. PMC10902350 38418445

[gcb70138-bib-0014] Biden, J. 2023. “Memorandum on Conserving the Natural and Cultural Heritage of the Pacific Remote Islands.” https://www.whitehouse.gov/briefing‐room/presidential‐actions/2023/03/24/memorandum‐on‐conserving‐the‐natural‐and‐cultural‐heritage‐of‐the‐pacific‐remote‐islands/.

[gcb70138-bib-0015] Blanluet, A. , E. T. Game , D. C. Dunn , J. D. Everett , A. T. Lombard , and A. J. Richardson . 2024. “Evaluating Ecological Benefits of Oceanic Protected Areas.” Trends in Ecology & Evolution 39, no. 2: 175–187. 10.1016/j.tree.2023.09.003. 37778906

[gcb70138-bib-0016] Boerder, K. , L. Schiller , and B. Worm . 2019. “Not all Who Wander Are Lost: Improving Spatial Protection for Large Pelagic Fishes.” Marine Policy 105: 80–90. 10.1016/j.marpol.2019.04.013.

[gcb70138-bib-0017] Brainard, R. E. , T. Acoba , M. Asher , et al. 2019. “Kingman Reef.” In Coral Reef Ecosystem Monitoring Report for the Pacific Remote Islands Marine National Monument 2000–2017, 89. PIFSC Special Publication. 10.25923/wb6x-c556.

[gcb70138-bib-0018] Braun, C. D. , M. C. Arostegui , N. Farchadi , et al. 2023. “Building Use‐Inspired Species Distribution Models: Using Multiple Data Types to Examine and Improve Model Performance.” Ecological Applications 33, no. 6: e2893. 10.1002/eap.2893. 37285072

[gcb70138-bib-0019] Calenge, C. 2006. “The Package Adehabitat for the R Software: A Tool for the Analysis of Space and Habitat Use by Animals. Version 0.4.21.” Ecological Modelling 197: 516–519.

[gcb70138-bib-0020] Carlisle, A. B. , D. Tickler , J. J. Dale , et al. 2019. “Estimating Space Use of Mobile Fishes in a Large Marine Protected Area With Methodological Considerations in Acoustic Array Design.” Frontiers in Marine Science 6: 00256. 10.3389/fmars.2019.00256.

[gcb70138-bib-0021] Caselle, J. E. , A. Rassweiler , S. L. Hamilton , and R. R. Warner . 2015. “Recovery Trajectories of Kelp Forest Animals Are Rapid Yet Spatially Variable Across a Network of Temperate Marine Protected Areas.” Scientific Reports 5: 14102. 10.1038/srep14102. 26373803 PMC4642697

[gcb70138-bib-0022] Collins, M. , S.‐I. An , W. Cai , et al. 2010. “The Impact of Global Warming on the Tropical Pacific Ocean and El Niño.” Nature Geoscience 3: 391–397. 10.1038/ngeo868.

[gcb70138-bib-0023] Conners, M. G. , N. B. Sisson , P. D. Agamboue , et al. 2022. “Mismatches in Scale Between Highly Mobile Marine Megafauna and Marine Protected Areas.” Frontiers in Marine Science 9: 897104. 10.3389/fmars.2022.897104.

[gcb70138-bib-0024] Curnick, D. , S. Andrzejaczek , D. M. P. Jacoby , et al. 2020a. “Behavior and Ecology of Silky Sharks Around the Chagos Archipelago and Evidence of Indian Ocean Wide Movement.” Frontiers in Marine Science 7: 596619. 10.3389/fmars.2020.596619.

[gcb70138-bib-0025] Curnick, D. , D. Feary , and G. Cavalcante . 2020b. “Risks to Large Marine Protected Areas Posed by Drifting Fish Aggregation Devices.” Conservation Biology 35: 1222–1232. 10.1111/cobi.13684. PMC8419855 33314325

[gcb70138-bib-0026] Dedman, S. , R. Officer , D. Brophy , M. Clarke , and D. G. Reid . 2017. “Advanced Spatial Modeling to Inform Management of Data‐Poor Juvenile and Adult Female Rays.” Fishes 2, no. 3: 12. 10.3390/fishes2030012.

[gcb70138-bib-0027] Donlon, C. , M. Martin , J. Stark , J. Roberts‐Jones , E. Fiedler , and W. Wimmer . 2012. “The Operational Sea Surface Temperature and Sea Ice Analysis (OSTIA) System.” Remote Sensing of Environment 116: 140–158. 10.1016/j.rse.2010.10.017.

[gcb70138-bib-0028] Douglas, D. C. , R. Weinzierl , S. C. Davidson , R. Kays , M. Wikelski , and G. Bohrer . 2012. “Moderating Argos Location Errors in Animal Tracking Data.” Methods in Ecology and Evolution 3, no. 6: 999–1007. 10.1111/j.2041-210X.2012.00245.x.

[gcb70138-bib-0029] Dunn, D. C. , A. L. Harrison , C. Curtice , et al. 2019. “The Importance of Migratory Connectivity for Global Ocean Policy.” Proceedings of the Royal Society B: Biological Sciences 286, no. 1911: 1472. 10.1098/rspb.2019.1472. PMC6784718 31551061

[gcb70138-bib-0030] E.U. Copernicus Marine Service Information (CMEMS) . 2025a. “Global Ocean Biogeochemistry Analysis and Forecast.” Marine Data Store. 10.48670/moi‐00015.

[gcb70138-bib-0031] E.U. Copernicus Marine Service Information (CMEMS) . 2025b. “Global Ocean Colour (Copernicus‐GlobColour), Bio‐Geo‐Chemical, L4 (Monthly and Interpolated) From Satellite Observations (1997‐ongoing).” Marine Data Store. 10.48670/moi‐00281.

[gcb70138-bib-0032] E.U. Copernicus Marine Service Information (CMEMS) . 2025c. “Global Ocean Physics Analysis and Forecast.” Marine Data Store. 10.48670/moi‐00016.

[gcb70138-bib-0033] Eddebbar, Y. A. , D. B. Whitt , A. Verdy , M. R. Mazloff , A. C. Subramanian , and M. C. Long . 2024. “Eddy‐Mediated Turbulent Mixing of Oxygen in the Equatorial Pacific.” Journal of Geophysical Research: Oceans 129, no. 3: 20588. 10.1029/2023JC020588.

[gcb70138-bib-0034] Eddy, T. D. , V. W. Y. Lam , G. Reygondeau , et al. 2021. “Global Decline in Capacity of Coral Reefs to Provide Ecosystem Services.” One Earth 4, no. 9: 1278–1285. 10.1016/j.oneear.2021.08.016.

[gcb70138-bib-0035] Eyring, V. , S. Bony , G. A. Meehl , et al. 2016. “Overview of the Coupled Model Intercomparison Project Phase 6 (CMIP6) Experimental Design and Organization.” Geoscientific Model Development 9, no. 5: 1937–1958. 10.5194/gmd-9-1937-2016.

[gcb70138-bib-0036] Filous, A. , A. M. Friedlander , M. Toribiong , R. J. Lennox , G. Mereb , and Y. Golbuu . 2022. “The Movements of Yellowfin Tuna, Blue Marlin, and Sailfish Within the Palau National Marine Sanctuary and the Western Pacific Ocean.” ICES Journal of Marine Science 79: 445–456. 10.1093/icesjms/fsac010.

[gcb70138-bib-0037] Fleishman, A. B. , R. A. Orben , and M. E. Gilmour . 2022. “Trakr: Basic Animal Tracking Data Analysis Tools.” 10.5281/zenodo.6588612. Version 0.0.12., https://github.com/abfleishman/trakR (Version 0.0.11) [Computer software].

[gcb70138-bib-0038] Fox, M. D. , R. Guillaume‐Castel , C. B. Edwards , et al. 2023. “Ocean Currents Magnify Upwelling and Deliver Nutritional Subsidies to Reef‐Building Corals During El Niño Heatwaves.” Science Advances 9, no. 24: eadd5032. 10.1126/sciadv.add5032. 37315146 PMC10266739

[gcb70138-bib-0039] Friedlander, A. , J. Arribas , E. Ballesteros , et al. 2017. “Exploring the Marine Ecosystems of Niue and Beveridge Reef.” In Report to the Government of Niue. National Geographic Pristine Seas.

[gcb70138-bib-0040] Friedlander, A. , S. A. Sandin , E. E. Demartini , and E. Sala . 2010. “Spatial Patterns of the Structure of Reef Fish Assemblages at a Pristine Atoll in the Central Pacific.” Marine Ecology Progress Series 410: 219–231. 10.3354/meps08634.

[gcb70138-bib-0041] Gilman, E. , M. Chaloupka , J. Peschon , and S. Ellgen . 2016. “Risk Factors for Seabird Bycatch in a Pelagic Longline Tuna Fishery.” PLoS One 11, no. 5: e0155477. 10.1371/journal.pone.0155477. 27192492 PMC4871550

[gcb70138-bib-0042] Gilmour, M. E. , J. Adams , B. Block , et al. 2024. “Palmyra Bluewater Research Marine Animal Telemetry Dataset 2022‐2023 [Dataset].” DataONE. 10.24431/rw1k8ez.

[gcb70138-bib-0043] Gilmour, M. E. , J. Adams , B. A. Block , et al. 2022. “Evaluation of MPA Designs That Protect Highly Mobile Megafauna Now and Under Climate Change Scenarios.” Global Ecology and Conservation 35: e02070. 10.1016/j.gecco.2022.e02070.

[gcb70138-bib-0044] Gilmour, M. E. , S. A. Trefry Hudson , C. Lamborg , A. B. Fleishman , H. S. Young , and S. A. Shaffer . 2019. “Tropical Seabirds Sample Broadscale Patterns of Marine Contaminants.” Science of the Total Environment 691: 631–643. 10.1016/j.scitotenv.2019.07.147. 31325863

[gcb70138-bib-0045] Good, S. , E. Fiedler , C. Mao , et al. 2020. “The Current Configuration of the OSTIA System for Operational Production of Foundation Sea Surface Temperature and Ice Concentration Analyses.” Remote Sensing 12, no. 4: 720. 10.3390/rs12040720.

[gcb70138-bib-0046] Gove, J. , M. A. McManus , A. B. Neuheimer , et al. 2016. “Near‐Island Biological Hotspots in Barren Ocean Basins.” Nature Communications 7: 10581. 10.1038/ncomms10581. PMC4757766 26881874

[gcb70138-bib-0047] Greenwell, B. , B. Boehmke , J. Cunningham , and GBM Developers . 2022. “gbm: Generalized boosted regression models.” R package version 2.1.8.1. https://CRAN.R‐project.org/package=gbm.

[gcb70138-bib-0048] Hausfather, Z. , K. Marvel , G. A. Schmidt , J. W. Nielsen‐Gammon , and M. Zelinka . 2022. “Climate Simulations: Recognize the ‘Hot Model’ Problem.” Nature 605, no. 7908: 26–29. 10.1038/d41586-022-01192-2. 35508771

[gcb70138-bib-0049] Hazen, E. L. , B. Abrahms , S. Brodie , G. Carroll , H. Welch , and S. J. Bograd . 2021. “Where Did They Not Go? Considerations for Generating Pseudo‐Absences for Telemetry‐Based Habitat Models.” Movement Ecology 9, no. 1: 5. 10.1186/s40462-021-00240-2. 33596991 PMC7888118

[gcb70138-bib-0050] Heede, U. K. , A. V. Fedorov , and N. J. Burls . 2020. “Time Scales and Mechanisms for the Tropical Pacific Response to Global Warming: A Tug of War Between the Ocean Thermostat and Weaker Walker.” Journal of Climate 33, no. 14: 6101–6118. 10.1175/JCLI-D-19-0690.1.

[gcb70138-bib-0051] Hernández, C. M. , J. Witting , C. Willis , S. R. Thorrold , J. K. Llopiz , and R. D. Rotjan . 2019. “Evidence and Patterns of Tuna Spawning Inside a Large No‐Take Marine Protected Area.” Scientific Reports 9, no. 1: 10772. 10.1038/s41598-019-47161-0. 31341251 PMC6656763

[gcb70138-bib-0052] Hijmans, R. J. , S. Phillips , J. Leathwick , and J. Elith . 2021. “Dismo: Species Distribution Modeling. R Packge Version 1.3–5.” https://CRAN.R‐project.org/package=dismo [R] https://CRAN.R‐project.org/package=dismo.

[gcb70138-bib-0053] Hill, M. C. , A. R. Bendlin , A. M. Van Cise , et al. 2019. “Short‐Finned Pilot Whales (*Globicephala Macrorhynchus*) of the Mariana Archipelago: Individual Affiliations, Movements, and Spatial Use.” Marine Mammal Science 35, no. 3: 797–824. 10.1111/mms.12567.

[gcb70138-bib-0054] Jacoby, D. M. P. , F. Ferretti , R. Freeman , et al. 2020. “Shark Movement Strategies Influence Poaching Risk and Can Guide Enforcement Decisions in a Large, Remote Marine Protected Area.” Journal of Applied Ecology 57, no. 9: 1782–1792. 10.1111/1365-2664.13654.

[gcb70138-bib-0055] Jonsen, I. D. , and T. Patterson . 2020. “Foiegras: Fit Latent Variable Movement Models to Animal Tracking Data for Location Quality Control and Behavioural Inference (Version 0.7‐6) [Computer Software].” 10.5281/zenodo.3899972.

[gcb70138-bib-0056] Khen, A. , M. D. Johnson , M. D. Fox , S. M. Clements , A. L. Carter , and J. E. Smith . 2022. “Decadal Stability of Coral Reef Benthic Communities on Palmyra Atoll, Central Pacific, Through Two Bleaching Events.” Coral Reefs 41, no. 4: 1017–1029. 10.1007/s00338-022-02271-6.

[gcb70138-bib-0057] Kim, H. , A. Timmermann , S. Lee , and F. Schloesser . 2023. “Rainfall and Salinity Effects on Future Pacific Climate Change.” Earth's Future 11, no. 8: e2022EF003457. 10.1029/2022EF003457.

[gcb70138-bib-0058] Kim, S.‐S. , and P. Wessel . 2011. “New Global Seamount Census From Altimetry‐Derived Gravity Data: New Global Seamount Census.” Geophysical Journal International 186, no. 2: 615–631. 10.1111/j.1365-246X.2011.05076.x.

[gcb70138-bib-0059] Kobayashi, D. R. 2006. “Colonization of the Hawaiian Archipelago via Johnston Atoll: A Characterization of Oceanographic Transport Corridors for Pelagic Larvae Using Computer Simulation.” Coral Reefs 25, no. 3: 407–417. 10.1007/s00338-006-0118-5.

[gcb70138-bib-0060] Lassauce, H. , O. Chateau , M. V. Erdmann , and L. Wantiez . 2020. “Diving Behavior of the Reef Manta Ray (Mobula Alfredi) in New Caledonia: More Frequent and Deeper Night‐Time Diving to 672 Meters.” PLoS One 15, no. 3: e0228815. 10.1371/journal.pone.0228815. 32187197 PMC7080230

[gcb70138-bib-0061] Lewis, N. , J. Day , A. Wilhelm , et al. 2017. “Large‐Scale Marine Protected Areas: Guidelines for design and management (BPG 26).” International Union for Conservation of Nature 26: 1–120. 10.2305/IUCN.CH.2017.PAG.26.en.

[gcb70138-bib-0062] Ling, M. , Z. Feng , Z. Chen , et al. 2024. “Evaluation of Driving Effects of Carbon Storage Change in the Source of the Yellow River: A Perspective With CMIP6 Future Development Scenarios.” Ecological Informatics 83: 102790. 10.1016/j.ecoinf.2024.102790.

[gcb70138-bib-0063] Madden, H. , Y. Satgé , B. Wilkinson , and P. G. R. Jodice . 2022. “Foraging Ecology of Red‐Billed Tropicbird *Phaethon Aethereus* in the Caribbean During Early Chick Rearing Revealed by GPS Tracking.” Marine Ornithology 50: 165–175.

[gcb70138-bib-0064] Magurran, A. E. , M. Dornelas , F. Moyes , N. J. Gotelli , and B. McGill . 2015. “Rapid Biotic Homogenization of Marine Fish Assemblages.” Nature Communications 6: 8405. 10.1038/ncomms9405. PMC4598618 26400102

[gcb70138-bib-0065] Maragos, J. , J. Miller , J. Gove , et al. 2008. “US Coral Reefs in the Line and Phoenix Islands, Central Pacific Ocean: History, Geology, Oceanography, and Biology.” In Coral Reefs of the USA, edited by B. M. Riegl and R. E. Dodge , 595–641. Springer Netherlands. 10.1007/978-1-4020-6847-8_15.

[gcb70138-bib-0066] Maxwell, S. M. , K. M. Gjerde , M. G. Conners , and L. B. Crowder . 2020. “Mobile Protected Areas for Biodiversity on the High Seas.” Science 367, no. 6475: 252–254. 10.1126/science.aaz9327. 31949070

[gcb70138-bib-0067] Maxwell, S. M. , E. L. Hazen , S. J. Bograd , et al. 2013. “Cumulative Human Impacts on Marine Predators.” Nature Communications 4, no. 1: 2688. 10.1038/ncomms3688. 24162104

[gcb70138-bib-0068] Maxwell, S. M. , and L. E. Morgan . 2013. “Foraging of Seabirds on Pelagic Fishes: Implications for Management of Pelagic Marine Protected Areas.” Marine Ecology Progress Series 481: 289–303. 10.3354/meps10255.

[gcb70138-bib-0069] McCauley, D. J. , P. A. DeSalles , H. S. Young , et al. 2012a. “From Wing to Wing: The Persistence of Long Ecological Interaction Chains in Less‐Disturbed Ecosystems.” Scientific Reports 2, no. 1: 409. 10.1038/srep00409. PMC3354671 22624091

[gcb70138-bib-0070] McCauley, D. J. , P. A. DeSalles , H. S. Young , et al. 2014. “Reliance of Mobile Species on Sensitive Habitats: A Case Study of Manta Rays ( *Manta alfredi* ) and Lagoons.” Marine Biology 161, no. 9: 1987–1998. 10.1007/s00227-014-2478-7.

[gcb70138-bib-0071] McCauley, D. J. , H. S. Young , R. B. Dunbar , J. A. Estes , B. X. Semmens , and F. Micheli . 2012b. “Assessing the Effects of Large Mobile Predators on Ecosystem Connectivity.” Ecological Applications 22, no. 6: 1711–1717. 10.1890/11-1653.1. 23092009

[gcb70138-bib-0072] Medoff, S. , J. Lynham , and J. Raynor . 2022. “Spillover Benefits From the world's Largest Fully Protected MPA.” Science 378, no. 6617: 313–316. 10.1126/science.abn0098. 36264800

[gcb70138-bib-0073] Millington, R. C. , A. Rogers , P. Cox , Y. Bozec , and P. J. Mumby . 2022. “Combined Direct and Indirect Impacts of Warming on the Productivity of Coral Reef Fishes.” Ecosphere 13, no. 7: e4108. 10.1002/ecs2.4108.

[gcb70138-bib-0074] Nelson, H. R. , and A. H. Altieri . 2019. “Oxygen: The Universal Currency on Coral Reefs.” Coral Reefs 38, no. 2: 177–198. 10.1007/s00338-019-01765-0.

[gcb70138-bib-0075] Olson, L. T. , and K. F. Lindsay . 2009. “Here Today, Gone Tomorrow? Targeting Conservation Investment in the Face of Climate Change.” Journal of Geography and Regional Planning 2, no. 1: 20–29.

[gcb70138-bib-0076] Pante, E. , and B. Simon‐Bouhet . 2013. “Marmap: A Package for Importing, Plotting and Analyzing Bathymetric and Topographic Data in R.” PLoS One 8, no. 9: e73051. 10.1371/journal.pone.0073051. 24019892 PMC3760912

[gcb70138-bib-0077] Pebesma, E. 2018. “Simple Features for R: Standardized Support for Spatial Vector Data. Version 1.0‐12.” R Journal 10, no. 1: 439–446. 10.32614/RJ-2018-009.

[gcb70138-bib-0078] Pedersen, M. , T. A. Patterson , U. Thygesen , and H. Madsen . 2011. “Estimating Animal Behavior and Residency From Movement Data.” Oikos 120: 1281–1290. 10.1111/j.1600-0706.2011.19044.x.

[gcb70138-bib-0079] Pendoley, K. L. , G. Schofield , P. A. Whittock , D. Ierodiaconou , and G. C. Hays . 2014. “Protected Species Use of a Coastal Marine Migratory Corridor Connecting Marine Protected Areas.” Marine Biology 161, no. 6: 1455–1466. 10.1007/s00227-014-2433-7.

[gcb70138-bib-0080] R Core Team . 2023. “R: A Language and Environment for Statistical Computing, Version 4.3.0. (Version 4.3.0) [Computer Software].R Foundation for Statistical Computing.” https://www.R‐project.org/.

[gcb70138-bib-0081] Raftery, A. E. , A. Zimmer , D. M. W. Frierson , R. Startz , and P. Liu . 2017. “Less Than 2°C Warming by 2100 Unlikely.” Nature Climate Change 7, no. 9: 637–641. 10.1038/nclimate3352. PMC6070153 30079118

[gcb70138-bib-0082] Sala, E. , J. Mayorga , D. Bradley , et al. 2021. “Protecting the Global Ocean for Biodiversity, Food and Climate.” Nature 592, no. 7854: 397–402. 10.1038/s41586-021-03371-z. 33731930

[gcb70138-bib-0083] Scales, K. L. , P. I. Miller , L. A. Hawkes , S. N. Ingram , D. W. Sims , and S. C. Votier . 2014. “On the Front Line: Frontal Zones as Priority At‐Sea Conservation Areas for Mobile Marine Vertebrates.” Journal of Applied Ecology 51, no. 6: 1575–1583. 10.1111/1365-2664.12330.

[gcb70138-bib-0084] Shea, B. D. , A. J. Gallagher , L. K. Bomgardner , and F. Ferretti . 2023. “Quantifying Longline Bycatch Mortality for Pelagic Sharks in Western Pacific Shark Sanctuaries.” Science Advances 9, no. 33: eadg3527. 10.1126/sciadv.adg3527. 37585534 PMC10431710

[gcb70138-bib-0085] Soanes, L. M. , J. A. Bright , G. Brodin , F. Mukhida , and J. A. Green . 2015. “Tracking a Small Seabird: First Records of Foraging Movements in the Sooty Tern *Onychoprion Fuscatus* .” Marine Ornithology 43: 235–239.

[gcb70138-bib-0086] Stark, J. , C. Donlon , M. Martin , and M. McCulloch . 2007. OSTIA: An Operational, High Resolution, Real Time, Global Sea Surface Temperature Analysis System. Marine Challenges: Coastline to Deep Sea. IEEE, Oceans, Aberdeen, Scotland.

[gcb70138-bib-0087] Stramma, L. , E. D. Prince , S. Schmidtko , et al. 2012. “Expansion of Oxygen Minimum Zones May Reduce Available Habitat for Tropical Pelagic Fishes.” Nature Climate Change 2, no. 1: 33–37. 10.1038/nclimate1304.

[gcb70138-bib-0088] Tolotti, M. T. , F. Forget , M. Capello , et al. 2020. “Association Dynamics of Tuna and Purse Seine Bycatch Species With Drifting Fish Aggregating Devices (FADs) in the Tropical Eastern Atlantic Ocean.” Fisheries Research 226: 105521. 10.1016/j.fishres.2020.105521.

[gcb70138-bib-0089] Tompkins, E. M. , H. M. Townsend , and D. J. Anderson . 2017. “Decadal‐Scale Variation in Diet Forecasts Persistently Poor Breeding Under Ocean Warming in a Tropical Seabird.” PLoS One 12, no. 8: e0182545. 10.1371/journal.pone.0182545. 28832597 PMC5568137

[gcb70138-bib-0090] U.S. Presidential Proclamation 8336 . 2009. “Establishment of the Pacific Remote Islands Marine National Monument.” https://www.federalregister.gov/documents/2009/01/12/E9‐500/establishment‐of‐the‐pacific‐remote‐islands‐marine‐national‐monument.

[gcb70138-bib-0091] U.S. Presidential Proclamation 9173 . 2014. “Proclamation 9173—Pacific Remote Islands Marine National Monument Expansion.” https://www.federalregister.gov/documents/2014/09/29/2014‐23319/pacific‐remote‐islands‐marine‐national‐monument‐expansion.

[gcb70138-bib-0092] United Nations . 2015. “Transforming Our World: The 2030 Agenda for Sustainable Development. Resolution Adopted by the General Assumbly on 25 September 2015. United Nations General Assembly.” https://sdgs.un.org/2030agenda.

[gcb70138-bib-0093] United Nations . 2023a. “Demonstrating ‘the Power of Multilateralism’, Intergovernmental Conference Adopts Historic New Maritime Biodiversity Treaty (A/CONF.232/2023/L.3).” https://press.un.org/en/2023/sea2181.doc.htm.

[gcb70138-bib-0094] United Nations . 2023b. “Draft Agreement Under the United Nations Convention on the Law of the Sea on the Conservation and Sustainable use of Marine Biological Diversity of Areas Beyond National Jurisdiction (A/CONF.232/2023/L.3; Intergovernmental Conference on an International Legally Binding Instrument Under the United Nations Convention on the Law of the Sea on the Conservation and Sustainable Use of Marine Biological Diversity of Areas Beyond National Jurisdiction.” https://documents.un.org/doc/undoc/ltd/n23/073/63/pdf/n2307363.pdf?token=tmzCRH3Fb25E2cWzap&fe=true.

[gcb70138-bib-0095] Valavi, R. , J. Elith , J. J. Lahoz‐Monfort , and G. Guillera‐Arroita . 2019. “Block CV: An r Package for Generating Spatially or Environmentally Separated Folds for k ‐Fold Cross‐Validation of Species Distribution Models.” Methods in Ecology and Evolution 10, no. 2: 225–232. 10.1111/2041-210X.13107.

[gcb70138-bib-0096] Visalli, M. E. , B. D. Best , R. B. Cabral , et al. 2020. “Data‐Driven Approach for Highlighting Priority Areas for Protection in Marine Areas Beyond National Jurisdiction.” Marine Policy 122: 103927. 10.1016/j.marpol.2020.103927.

[gcb70138-bib-0097] Weimerskirch, H. , and A. Prudor . 2019. “Cyclone Avoidance Behaviour by Foraging Seabirds.” Scientific Reports 9, no. 1: 5400. 10.1038/s41598-019-41481-x. 30931969 PMC6443659

[gcb70138-bib-0098] Welch, H. , M. S. Savoca , S. Brodie , et al. 2023. “Impacts of Marine Heatwaves on Top Predator Distributions Are Variable but Predictable.” Nature Communications 14, no. 1: 5188. 10.1038/s41467-023-40849-y. PMC10480173 37669922

[gcb70138-bib-0099] White, T. D. , A. B. Carlisle , D. A. Kroodsma , et al. 2017. “Assessing the Effectiveness of a Large Marine Protected Area for Reef Shark Conservation.” Biological Conservation 207: 64–71. 10.1016/j.biocon.2017.01.009.

[gcb70138-bib-0100] Wilson, S. G. , I. D. Jonsen , R. J. Schallert , et al. 2015. “Tracking the Fidelity of Atlantic Bluefin Tuna Released in Canadian Waters to the Gulf of Mexico Spawning Grounds.” Canadian Journal of Fisheries and Aquatic Sciences 72, no. 11: 1700–1717. 10.1139/cjfas-2015-0110.

[gcb70138-bib-0101] Wood, S. N. 2011. “Fast Stable Restricted Maximum Likelihood and Marginal Likelihood Estimation of Semiparametric Generalized Linear Models.” Journal of the Royal Statistical Society (B) 73, no. 1: 3–36.

[gcb70138-bib-0102] You, K. 2022. “Riemann: Learning with data on Riemannian manifolds. R package version 0.1.4.” https://CRAN.R‐project.org/package=Riemann (Version 0.1.4) [R]. https://CRAN.R‐project.org/package=Riemann.

[gcb70138-bib-0103] Young, H. S. , S. M. Maxwell , M. G. Conners , and S. A. Shaffer . 2015. “Pelagic Marine Protected Areas Protect Foraging Habitat for Multiple Breeding Seabirds in the Central Pacific.” Biological Conservation 181: 226–235. 10.1016/j.biocon.2014.10.027.

[gcb70138-bib-0104] Young, H. S. , K. Nigro , D. J. McCauley , L. T. Ballance , E. M. Oleson , and S. Baumann‐Pickering . 2017. “Limited Trophic Partitioning Among Sympatric Delphinids Off a Tropical Oceanic Atoll.” PLoS One 12, no. 8: e0181526. 10.1371/journal.pone.0181526. 28767677 PMC5540553

