# Supplementary material for: Multi‐Species Telemetry Quantifies Current and Future Efficacy of a Remote Marine Protected Area
Source: Glob Chang Biol. 2025 Apr 15;31(4):e70138. doi: 10.1111/gcb.70138 (PMC11997735; doi:10.1111/gcb.70138)
Supplement: Supplementary file 1 — Data S1 Supporting Information. [file GCB-31-e70138-s001.pdf]

Supplemental material for

Multi-species telemetry quantifies current and future efficacy of a remote marine protected area

M. E. Gilmour*, K. Pollock, J. Adams, B. A. Block, J. E. Caselle, A. Filous, A. M. Friedlander, E. T. Game, E. L. Hazen, M. Hill, N. D. Holmes, K. D. Lafferty, S. M. Maxwell, D. J. McCauley, R. Schallert, S. A. Shaffer, N. H. Wolff, A. Wegmann

* Corresponding author: Morgan Gilmour, morgan.e.gilmour@nasa.gov

Any use of trade, firm, or product names is for descriptive purposes only and does not imply endorsement by the U.S. Government.

This supplemental material contains the following:

Text S1: Brief description of species distribution modeling approach results.

Table S1: Summary of GPE3 model scores.

Table S2: Predictive skill statistics of species distribution modeling approaches.

Table S3: Area under the curve calculated for boosted regression tree models and cross validation approaches.

Table S4: Climate models used in habitat suitability models under climate scenarios.

Table S5: Relative influence of environmental variables in species distribution models.

Table S6: Median habitat suitability and percent change in habitat suitability under climate change scenarios.

Table S7: Predicted changes in habitat suitability with the MPA and US EEZ surrounding Palmyra.

Figure S1: Boxplots of cetacean and fishes' dive depths.

Figure S2: Partial effects plots of species distribution models.

Figure S3: Habitat suitability for each species predicted for summer.

Figure S4: Habitat suitability for each species predicted for winter.

Figure S5: Predicted change in habitat suitability under two climate scenarios and two time periods for summer.

Figure S6: Habitat suitability for reef manta ray, calculated for three model types and three pseudo-absence types.

Figure S7: Habitat suitability for grey reef shark, calculated for three model types and three pseudo-absence types.

Figure S8: Habitat suitability for yellowfin tuna, calculated for three model types and three pseudo-absence types.

Figure S9: Habitat suitability for sooty tern, calculated for three model types and three pseudo-absence types.

Figure S10: Habitat suitability for great frigatebird, calculated for three model types and three pseudo-absence types.

Figure S11: Habitat suitability for red-footed booby, calculated for three model types and three pseudo-absence types.

Figure S12: Habitat suitability for melon-headed whale, calculated for three model types and three pseudo-absence types.

Figure S13: Habitat suitability for bottlenose dolphin, calculated for three model types and three pseudo-absence types.

Text S1

Species distribution modeling approaches: Models that used background sampling and boosted regression trees consistently returned the best model fits and had the highest predictive skill for all species except sooty terns (**Table S2, S3**). The best fitting models resulted in distinct regions of high (>0.67) and low (<0.3) habitat suitability, whereas poorer fitting models corresponded to moderate habitat suitability (0.5) that was relatively homogenous throughout the model extents (**Figures S6–S13**). Homogenous moderate habitat suitability was consistently prominent among the CRW pseudo-absences, especially when combined with GLMMs. Two temporal resolutions were used to construct CRW pseudo-absences: daily median locations and CRW at the original resolution of the tag; the latter approach enabled larger sample sizes. However, the original resolution-CRW resulted in more homogenous moderate habitat suitability than the daily resolution-CRW, and model results were similar between BRT, GAMM, and GLMM within this pseudo-absence type (e.g., **Figure S10**). Environmental dissimilarity, represented by Bhattacharyya coefficients, was slightly higher for CRW pseudo-absences than background pseudo-absences (**Table S2**). These differences were relatively small, and visually, the

contrast between high and low habitat suitability was most distinct for background pseudo-absences (**Figures S6–S13**).

Table S1

Table S1: Summary of Global Position Estimator 3 (GPE3) model scores and associated speeds for Wildlife Computers MiniPAT-348 tags. Dash indicates speed threshold was not tested for that species.

	**Mean (± SD) model score**			
**Species**	Blue marlin	Grey reef shark	Reef manta ray	Yellowfin tuna
**Speed threshold (m s^{-1})**	(n=1)	(n=6)	(n=4)	(n=9)
0.5	–	77.6 ± 3.8	–	–
0.75	–	78.7 ± 3.1	–	–
1.0	–	78.3 ± 3.0	–	51.3 ± 12.2
1.25	–	77.0 ± 4.3	–	–
1.5	77.0	77.0 ± 4.2	50.1 ± 16.6	55.1 ± 11.2
1.75	–	77.1 ± 4.0	–	–
2.0	77.9	77.0 ± 3.8	50.6 ± 16.5	55.8 ± 11.4
2.25	–	77.0 ± 3.7	–	–
2.5	78.0	76.9 ± 3.7	51.7 ± 16.0	55.9 ± 12.5
2.75	–	76.9 ± 3.6	–	–
3.0	78.0	76.8 ± 3.6	52.4 ± 15.5	56.9 ± 11.7

Table S2

Table S2: Model predictive skill statistics summary. The top three model metrics per species are bolded. Abbreviations: PA: pseudo-absence; BA: Bhattacharyya coefficient; AUC: area under the receiver operator curve; TSS: true test statistic; BRT: boosted regression tree; GAMM: generalized additive mixed model; GLMM: generalized linear mixed model.

Species	PA-type	Temporal res.	Mean BA	Model type	R^2	AUC	TSS
Reef manta ray	Background	Daily	0.90	BRT	**0.79**	**0.91**	**0.77**
				GAMM	0.41	0.88	0.68
				GLMM	0.34	0.86	0.63
	CRW	Original	0.93	BRT	**0.72**	**0.90**	**0.69**
				GAMM	0.30	0.79	0.53
				GLMM	0.13	0.69	0.40
	CRW	Daily	0.92	BRT	**0.79**	**0.95**	**0.83**
				GAMM	0.41	0.86	0.63
				GLMM	0.22	0.78	0.53
Grey reef shark	Background	Daily	0.86	BRT	**0.65**	**0.91**	**0.76**
				GAMM	**0.63**	**0.95**	**0.78**
				GLMM	**0.47**	**0.91**	**0.72**
	CRW	Original	0.96	BRT	0.33	0.80	0.47
				GAMM	0.19	0.77	0.42
				GLMM	0.09	0.69	0.31
	CRW	Daily	0.96	BRT	0.39	0.82	0.52
				GAMM	0.17	0.74	0.39
				GLMM	0.08	0.68	0.30
Yellowfin tuna	Background	Daily	0.91	BRT	**0.66**	**0.89**	**0.70**
				GAMM	0.34	**0.84**	**0.59**
				GLMM	0.21	0.78	0.52
	CRW	Original	0.94	BRT	**0.53**	0.83	0.55
				GAMM	0.28	0.80	0.50
				GLMM	0.10	0.69	0.36
	CRW	Daily	0.93	BRT	**0.58**	**0.87**	**0.62**
				GAMM	0.24	0.77	0.51
				GLMM	0.14	0.72	0.41
Sooty tern	Background	Daily	0.87	BRT	**0.71**	0.44	0.29
				GAMM	0.13	0.42	0.32

				GLMM	0.09	0.48	0.36
	CRW	Original	0.88	BRT	**0.65**	0.41	0.25
				GAMM	0.10	0.54	0.42
				GLMM	0.10	0.58	0.42
	CRW	Daily	0.84	BRT	**0.71**	**0.67**	**0.55**
				GAMM	0.29	**0.59**	**0.47**
				GLMM	0.25	**0.65**	**0.48**
Red-footed booby	Background	Daily	0.80	BRT	**0.81**	**0.90**	**0.76**
				GAMM	0.54	**0.88**	**0.74**
				GLMM	0.34	**0.85**	**0.63**
	CRW	Original	0.94	BRT	**0.58**	0.78	0.52
				GAMM	0.20	0.78	0.54
				GLMM	0.20	0.78	0.54
	CRW	Daily	0.91	BRT	**0.71**	0.79	0.55
				GAMM	0.32	0.81	0.55
				GLMM	0.19	0.74	0.47
Great frigatebird	Background	Daily	0.89	BRT	**0.67**	**0.90**	**0.70**
				GAMM	0.46	0.88	0.67
				GLMM	0.36	0.86	0.62
	CRW	Original	0.98	BRT	0.27	0.60	0.23
				GAMM	0.05	0.62	0.28
				GLMM	0.04	0.62	0.28
	CRW	Daily	0.84	BRT	**0.71**	**0.92**	**0.70**
				GAMM	**0.54**	**0.91**	**0.70**
				GLMM	0.36	0.86	0.60
Melon-headed whale	Background	Daily	0.78	BRT	**0.95**	**0.95**	**0.86**
				GAMM	0.73	**0.94**	**0.83**
				GLMM	0.68	**0.94**	**0.86**
	CRW	Original	0.90	BRT	**0.77**	0.86	0.72
				GAMM	0.32	0.79	0.57
				GLMM	0.31	0.79	0.57
	CRW	Daily	0.86	BRT	**0.86**	0.93	0.82
				GAMM	0.61	0.90	0.76
				GLMM	0.48	0.90	0.78
Bottlenose dolphin	Background	Daily	0.73	BRT	**0.99**	**0.99**	**0.94**
				GAMM	**0.99**	**0.97**	**0.91**

				GLMM	**0.99**	**0.98**	**0.93**
	CRW	Original	0.92	BRT	0.48	0.52	0.30
				GAMM	0.19	0.66	0.46
				GLMM	0.06	0.47	0.28
	CRW	Daily	0.93	BRT	0.59	0.53	0.26
				GAMM	0.19	0.80	0.64
				GLMM	0.12	0.58	0.36

Table S3

Table S3: Area under the curve (AUC) values from the receiver operator curves for the boosted regression tree (BRT) model and the mean AUC from each of the cross validation (CV) approaches.

Species	AUC		
	BRT model	10-fold CV	Spatial block CV
Reef manta ray	0.91	0.91	0.75
Grey reef shark	0.89	0.91	0.82
Yellowfin tuna	0.87	0.89	0.80
Sooty tern	0.65	0.44	0.85
Red-footed booby	0.93	0.90	0.89
Great frigatebird	0.86	0.90	0.83
Melon-headed whale	0.95	0.95	0.89
Bottlenose dolphin	0.99	0.99	0.89

Table S4

Table S4: Climate models used in habitat suitability models with climate predictions from the Climate Model Intercomparison Project Phase 6 (CMIP6). Model names are structured as follows: (mip_era)s.%(activity_drs)s.%(institution_id)s.%(source_id)s.%(experiment_id)s.%(member_id)s.%(table_id)s.%(variable_id)s.%(grid_label)s. Within each model/experiment, all available members (which typically had different starting parameters) were used.

Variable	Variable ID	Climate scenario	Model (experiment) name	DOI
Chlorophyll-*a*	chlpico	SSP-1-2.6	CMIP.NCAR.CESM2-FV2.historical.Omon.gr	https://doi.org/10.22033/ESGF/CMIP6.11297
			CMIP.NCAR.CESM2.historical.Omon.gr	https://doi.org/10.22033/ESGF/CMIP6.7627
			CMIP.NCAR.CESM2-WACCM-FV2.historical.Omon.gr	https://doi.org/10.22033/ESGF/CMIP6.11298
			CMIP.NCAR.CESM2-WACCM.historical.Omon.gr	https://doi.org/10.22033/ESGF/CMIP6.10071
			ScenarioMIP.NCAR.CESM2-WACCM.ssp126.Omon.gr	https://doi.org/10.22033/ESGF/CMIP6.10100
Chlorophyll-*a*	chlpico	SSP-3-7.0	CMIP.NCAR.CESM2-FV2.historical.Omon.gr	https://doi.org/10.22033/ESGF/CMIP6.11297
			CMIP.NCAR.CESM2.historical.Omon.gr	https://doi.org/10.22033/ESGF/CMIP6.7627
			CMIP.NCAR.CESM2-WACCM-FV2.historical.Omon.gr	https://doi.org/10.22033/ESGF/CMIP6.11298
			CMIP.NCAR.CESM2-WACCM.historical.Omon.gr	https://doi.org/10.22033/ESGF/CMIP6.10071
			ScenarioMIP.NCAR.CESM2-WACCM.ssp370.Omon.gr	https://doi.org/10.22033/ESGF/CMIP6.10102
Dissolved oxygen	o2sat	SSP-1-2.6	CMIP.NCC.NorESM2-LM.historical.Omon.gr	https://doi.org/10.22033/ESGF/CMIP6.8036
			CMIP.NCC.NorESM2-MM.historical.Omon.gr	https://doi.org/10.22033/ESGF/CMIP6.8040
			CMIP.NOAA-GFDL.GFDL-ESM4.historical.Omon.gr	https://doi.org/10.22033/ESGF/CMIP6.8597
			ScenarioMIP.NOAA-GFDL.GFDL-ESM4.ssp126.Omon.gr	https://doi.org/10.22033/ESGF/CMIP6.8684
Dissolved oxygen	o2sat	SSP-3-7.0	CMIP.NCC.NorESM2-LM.historical.Omon.gr	https://doi.org/10.22033/ESGF/CMIP6.8036
			CMIP.NCC.NorESM2-MM.historical.Omon.gr	https://doi.org/10.22033/ESGF/CMIP6.8040
			CMIP.NOAA-GFDL.GFDL-ESM4.historical.Omon.gr	https://doi.org/10.22033/ESGF/CMIP6.8597
			ScenarioMIP.NCC.NorESM2-LM.ssp370.Omon.gr	https://doi.org/10.22033/ESGF/CMIP6.8268
			ScenarioMIP.NCC.NorESM2-MM.ssp370.Omon.gr	https://doi.org/10.22033/ESGF/CMIP6.8270
			ScenarioMIP.NOAA-GFDL.GFDL-ESM4.ssp370.Omon.gr	https://doi.org/10.22033/ESGF/CMIP6.8691
SST	tos	SSP-3-7.0	CMIP.E3SM-Project.E3SM-1-0.historical.Omon.gr	https://doi.org/10.22033/ESGF/CMIP6.4497
			CMIP.E3SM-Project.E3SM-1-1.historical.Omon.gr	https://doi.org/10.22033/ESGF/CMIP6.11485
			CMIP.E3SM-Project.E3SM-1-1-ECA.historical.Omon.gr	https://doi.org/10.22033/ESGF/CMIP6.11486
			CMIP.EC-Earth-Consortium.EC-Earth3-Veg.historical.Omon.gr	https://doi.org/10.22033/ESGF/CMIP6.4706
			CMIP.MRI.MRI-ESM2-0.historical.Omon.gr	https://doi.org/10.22033/ESGF/CMIP6.6842
			CMIP.NCAR.CESM2-FV2.historical.Omon.gr	https://doi.org/10.22033/ESGF/CMIP6.11297

			CMIP.NCAR.CESM2.historical.Omon.gr	https://doi.org/10.22033/ESGF/CMIP6.7627
			CMIP.NCAR.CESM2-WACCM.historical.Omon.gr	https://doi.org/10.22033/ESGF/CMIP6.10071
			CMIP.NCAR.CESM2-WACCM-FV2.historical.Omon.gr	https://doi.org/10.22033/ESGF/CMIP6.11298
			CMIP.NIMS-KMA.KACE-1-0-G.historical.Omon.gr	https://doi.org/10.22033/ESGF/CMIP6.8378
			CMIP.NOAA-GFDL.GFDL-CM4.historical.Omon.gr	https://doi.org/10.22033/ESGF/CMIP6.8594
			CMIP.NOAA-GFDL.GFDL-ESM4.historical.Omon.gr	https://doi.org/10.22033/ESGF/CMIP6.8597
			ScenarioMIP.MRI.MRI-ESM2-0.ssp370.Omon.gr	https://doi.org/10.22033/ESGF/CMIP6.6915
			ScenarioMIP.NCAR.CESM2-WACCM.ssp370.Omon.gr	https://doi.org/10.22033/ESGF/CMIP6.10102
			ScenarioMIP.NIMS-KMA.KACE-1-0-G.ssp370.Omon.gr	https://doi.org/10.22033/ESGF/CMIP6.8437
			ScenarioMIP.NOAA-GFDL.GFDL-ESM4.ssp370.Omon.gr	https://doi.org/10.22033/ESGF/CMIP6.8691
SST	tos	SSP-1-2.6	CMIP.E3SM-Project.E3SM-1-0.historical.Omon.gr	https://doi.org/10.22033/ESGF/CMIP6.4497
			CMIP.E3SM-Project.E3SM-1-1.historical.Omon.gr	https://doi.org/10.22033/ESGF/CMIP6.11485
			CMIP.E3SM-Project.E3SM-1-1-ECA.historical.Omon.gr	https://doi.org/10.22033/ESGF/CMIP6.11486
			CMIP.EC-Earth-Consortium.EC-Earth3-Veg.historical.Omon.gr	https://doi.org/10.22033/ESGF/CMIP6.4706
			CMIP.MRI.MRI-ESM2-0.historical.Omon.gr	https://doi.org/10.22033/ESGF/CMIP6.6842
			CMIP.NCAR.CESM2-FV2.historical.Omon.gr	https://doi.org/10.22033/ESGF/CMIP6.11297
			CMIP.NCAR.CESM2.historical.Omon.gr	https://doi.org/10.22033/ESGF/CMIP6.7627
			CMIP.NCAR.CESM2-WACCM.historical.Omon.gr	https://doi.org/10.22033/ESGF/CMIP6.10071
			CMIP.NCAR.CESM2-WACCM-FV2.historical.Omon.gr	https://doi.org/10.22033/ESGF/CMIP6.11298
			CMIP.NIMS-KMA.KACE-1-0-G.historical.Omon.gr	https://doi.org/10.22033/ESGF/CMIP6.8378
			CMIP.NOAA-GFDL.GFDL-CM4.historical.Omon.gr	https://doi.org/10.22033/ESGF/CMIP6.8594
			CMIP.NOAA-GFDL.GFDL-ESM4.historical.Omon.gr	https://doi.org/10.22033/ESGF/CMIP6.8597
			ScenarioMIP.MRI.MRI-ESM2-0.ssp126.Omon.gr	https://doi.org/10.22033/ESGF/CMIP6.6909
			ScenarioMIP.NCAR.CESM2-WACCM.ssp126.Omon.gr	https://doi.org/10.22033/ESGF/CMIP6.10100
			ScenarioMIP.NIMS-KMA.KACE-1-0-G.ssp126.Omon.gr	https://doi.org/10.22033/ESGF/CMIP6.8432
			ScenarioMIP.NOAA-GFDL.GFDL-ESM4.ssp126.Omon.gr	https://doi.org/10.22033/ESGF/CMIP6.8684
Surface currents: u, v	uo, vo	SSP-1-2.6	CMIP.E3SM-Project.E3SM-1-1-ECA.historical.Omon.gr	https://doi.org/10.22033/ESGF/CMIP6.11486
			CMIP.E3SM-Project.E3SM-1-1.historical.Omon.gr	https://doi.org/10.22033/ESGF/CMIP6.11485
			CMIP.NCC.NorESM2-LM.historical.Omon.gr	https://doi.org/10.22033/ESGF/CMIP6.8036
			ScenarioMIP.NCC.NorESM2-LM.ssp126.Omon.gr	https://doi.org/10.22033/ESGF/CMIP6.8248
Surface currents: u, v	uo, vo	SSP-3-7.0	CMIP.E3SM-Project.E3SM-1-1.historical.Omon.gr	https://doi.org/10.22033/ESGF/CMIP6.11486
			CMIP.E3SM-Project.E3SM-1-1-ECA.historical.Omon.gr	https://doi.org/10.22033/ESGF/CMIP6.11485
			CMIP.NASA-GISS.GISS-E2-1-H.historical.Omon.gr	https://doi.org/10.22033/ESGF/CMIP6.7128

			CMIP.NCC.NorESM2-LM.historical.Omon.gr	https://doi.org/10.22033/ESGF/CMIP6.8036
			ScenarioMIP.NCC.NorESM2-LM.ssp370.Omon.gr	https://doi.org/10.22033/ESGF/CMIP6.8268

Table S5

Table S5: Relative influence (%) of environmental variables in species distribution models. Bolded values indicate relative influence >10%.

Primary habitat group	Species	Bathymetric depth	Dissolved O_2	SST	Chl-*a*	Surface current velocity	Surface current heading
Reef–pelagic	Reef manta ray	**46.2**	**22.3**	6.8	7.8	5.9	**11.0**
	Grey reef shark	**76.9**	4.2	2.8	9.0	2.7	4.3
Pelagic	Yellowfin tuna	**52.1**	**12.2**	**11.0**	**11.6**	5.1	8.0
	Sooty tern	**18.9**	**21.1**	**10.7**	**18.2**	**17.6**	**13.6**
	Great frigatebird	**54.5**	7.8	**12.8**	**10.5**	4.4	9.9
Nearshore–pelagic	Bottlenose dolphin	**93.4**	2.1	0.7	0.7	1.1	2.1
	Melon-headed whale	**69.2**	6.3	2.5	7.7	5.7	8.6
	Red-footed booby	**57.3**	5.3	6.3	6.9	**11.6**	**12.5**

Abbreviations: Chl-*a*, chlorophyll-*a*; SST, sea surface temperature.

Table S6

Table S6: Predicted median habitat suitability (HS) and percent change in HS under two climate scenarios at Palmyra. Percent change in HS between historic (1984–2014) and projected time periods (2040–2050; 2090–2100) are listed in parentheses with +/- indicating the direction of the change. The percentage of grid cells with highly suitable habitat (HS >0.67) within the marine protected area (MPA) and U.S. exclusive economic zone (EEZ) are also provided. The mean change per habitat group is also shown.

			**Habitat suitability 1984–2014**				**2040–2050**				**2090–2100**			
			**MPA**		**EEZ**		**MPA**		**EEZ**		**MPA**		**EEZ**	
	**Scenario**		**1-2.6**	**3-7.0**	**1-2.6**	**3-7.0**	**1-2.6**	**3-7.0**	**1-2.6**	**3-7.0**	**1-2.6**	**3-7.0**	**1-2.6**	**3-7.0**
**Primary habitat**	**Species**													
Reef-pelagic	Reef manta ray	Median HS	0.53	0.82	0.53	0.62	0.26	0.69	0.69	0.67	0.71	0.92	0.63	0.70
		% Change in HS	–	–	–	–	(-51%)	(-16%)	(+30%)	(+8%)	(+34%)	(+12%)	(+19%)	(+13%)
		% Highly suitable habitat	0%	79%	31%	45%	0	54%	50%	5%	68%	91%	49%	60%
	Grey reef shark	Median HS	0.11	0.12	0.05	0.06	0.02	0.06	0.05	0.03	0.04	0.05	0.06	0.17
		% Change in HS	–	–	–	–	(-82%)	(-50%)	(0%)	(-50%)	(-64%)	(-58%)	(+20%)	(+183%)
		% Highly suitable habitat	0%	0%	16%	15%	0	0	0	13%	0	0	10%	0.2%
	**Mean % change in HS**						**-66%**	**-33%**	**+15%**	**-21%**	**-15%**	**-23%**	**+20%**	**+98%**
Pelagic	Yellowfin tuna	Median HS	0.87	0.96	0.86	0.86	0.85	0.93	0.80	0.82	0.92	0.96	0.84	0.86
		% Change in HS	–	–	–	–	(-2%)	(-3%)	(-7%)	(-5%)	(+6%)	(0%)	(-2%)	(0%)
		% Highly suitable habitat	100%	100%	96%	93%	100%	98%	78%	83%	100%	100%	74%	90%
	Sooty tern	Median HS	0.83	0.48	0.75	0.34	0.73	0.30	0.88	0.30	0.81	0.30	0.80	0.32
		% Change in HS	–	–	–	–	(-12%)	(-38%)	(+17%)	(-12%)	(-2%)	(-38%)	(+7%)	(-6%)
		% Highly suitable habitat	100%	2%	65%	8%	100%	0	94%	2%	100%	0	81%	1%
	Great frigatebird	Median HS	0.81	0.87	0.73	0.70	0.77	0.18	0.20	0.14	0.85	0.82	0.64	0.60
		% Change in HS	–	–	–	–	(-5%)	(-79%)	(-73%)	(-80%)	(+5%)	(-6%)	(-12%)	(-14%)
		% Highly suitable habitat	83%	92%	%55	55%	89%	37%	21%	23%	98%	81%	47%	44%
	**Mean % change in HS**						**-6%**	**-40%**	**-21%**	**-32%**	**+3%**	**-15%**	**-2%**	**-7%**
Nearshore-pelagic	Red-footed booby	Median HS	0.80	0.51	0.43	0.45	0.39	0.20	0.44	0.35	0.73	0.44	0.43	0.43
		% Change in HS	–	–	–	–	(-51%)	(-61%)	(+2%)	(-22%)	(-9%)	(-14%)	(0%)	(-4%)
		% Highly suitable habitat	75%	18%	27%	26%	18%	11%	20%	15%	94%	10%	33%	21%

Table S7

Table S7: Mean (± SD) predicted change in habitat suitability within the marine protected area (MPA) and U.S. exclusive economic zone (EEZ) surrounding Palmyra-Kingman between the historic model and each decade of interest for each climate scenario. Mean changes >0.2 are bolded. Habitat suitability was estimated with background samples and boosted regression trees.

		**Change in habitat suitability**							
		**Historical: 2040–2050**				**Historical: 2090–2100**			
		**MPA**		**EEZ**		**MPA**		**EEZ**	
**Primary habitat**	**Scenario** / **Species**	**1-2.6**	**3-7.0**	**1-2.6**	**3-7.0**	**1-2.6**	**3-7.0**	**1-2.6**	**3-7.0**
Reef-pelagic	Reef manta ray	-0.03 ± 0.28	-0.07 ± 0.20	0.16 ± 0.32	0.05 ± 0.16	**0.21 ± 0.15**	0.09 ± 0.10	0.10 ± 0.28	0.08 ± 0.13
	Grey reef shark	-0.10 ± 0.10	-0.04 ± 0.04	-0.15 ± 0.32	-0.04 ± 0.14	-0.08 ± 0.12	-0.03 ± 0.08	-0.04 ± 0.45	-0.02 ± 0.31
	Mean change	-0.04	-0.06	+0.01	+0.01	+0.07	+0.03	+0.03	+0.03
Pelagic	Yellowfin tuna	-0.02 ± 0.07	-0.04 ± 0.05	-0.08 ± 0.13	-0.04 ± 0.07	0.04 ± 0.02	-0.02 ± 0.02	-0.08 ± 0.17	-0.01 ± 0.08
	Sooty tern	-0.04 ± 0.04	-0.06 ± 0.22	0.15 ± 0.16	-0.07 ± 0.25	-0.01 ± 0.07	-0.02 ± 0.16	0.06 ± 0.21	-0.05 ± 0.21
	Great frigatebird	-0.03 ± 0.13	**-0.38 ± 0.32**	**-0.24 ± 0.28**	**-0.26 ± 0.30**	0.03 ± 0.11	-0.01 ± 0.13	-0.04 ± 0.36	6.6×10^{-5} ± 0.28
	Mean change	-0.03	-0.16	-0.06	-0.12	0.02	-0.02	-0.02	-0.02
Nearshore-pelagic	Red-footed booby	**-0.29 ± 0.29**	-0.19 ± 0.14	-0.02 ± 0.25	-0.06 ± 0.16	0.03 ± 0.14	-0.09 ± 0.27	0.01 ± 0.21	0.03 ± 0.32

Figure S1

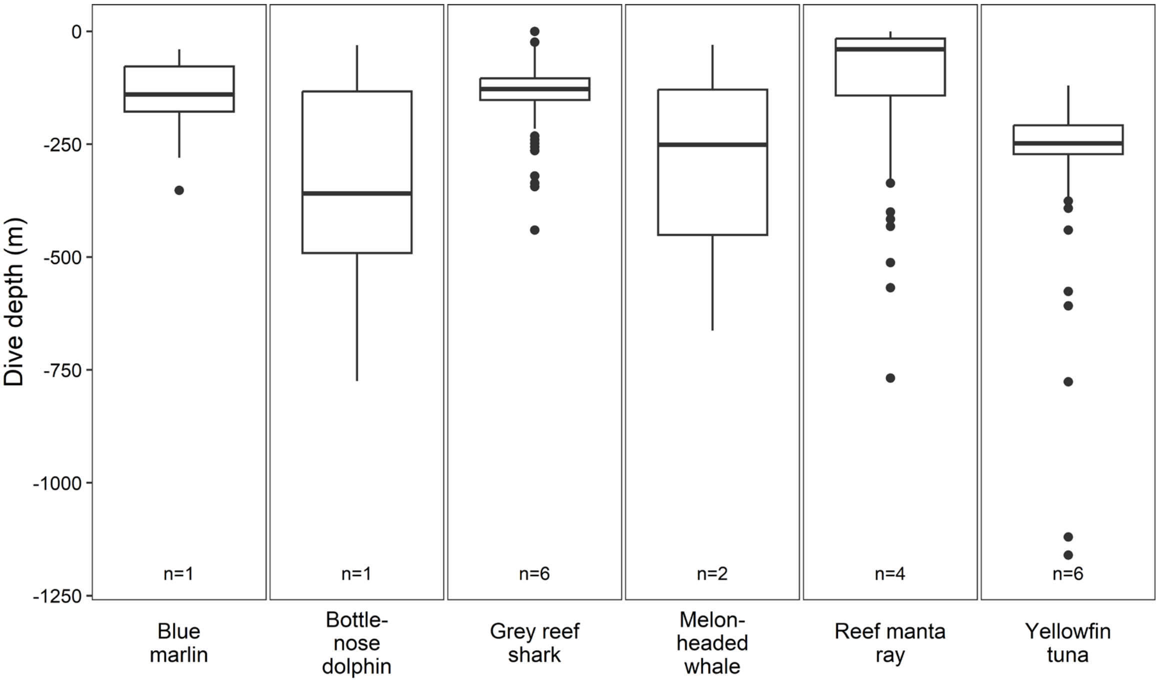

Figure S1: Water column use by cetaceans and fishes. Boxplots represent maximum dive depth (m) recorded per day per individual, grouped by species. Individual points represent observations that occurred outside the interquartile range and horizontal line within boxes are the mean maximum daily depth. Sample sizes are provided within each panel.

Figure S2

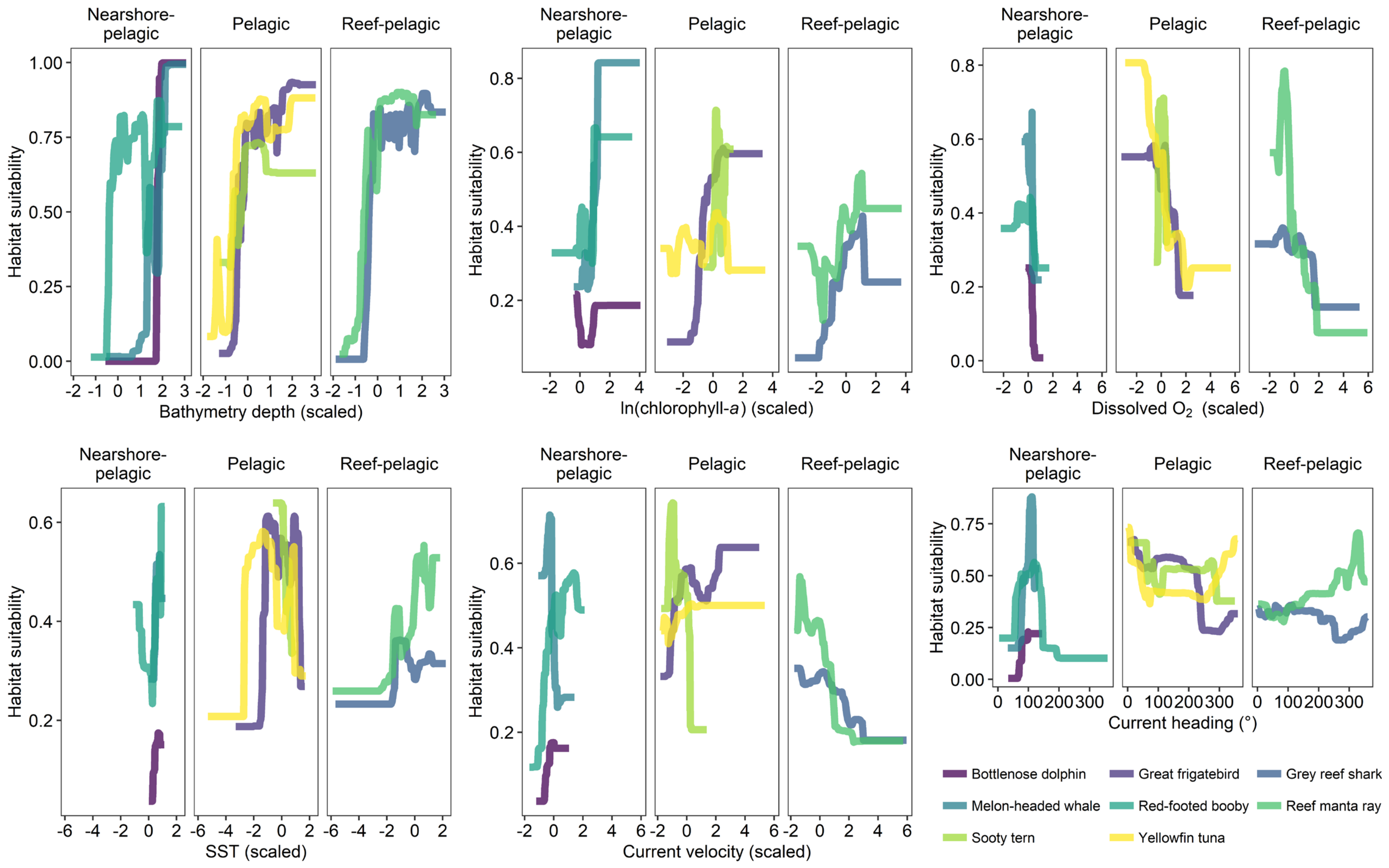

Figure S2: Partial effects plots of each environmental variable on habitat suitability from boosted regression trees conducted with background pseudo-absences. All variables except current heading were centered and scaled before analyses and are unitless.

Figure S3

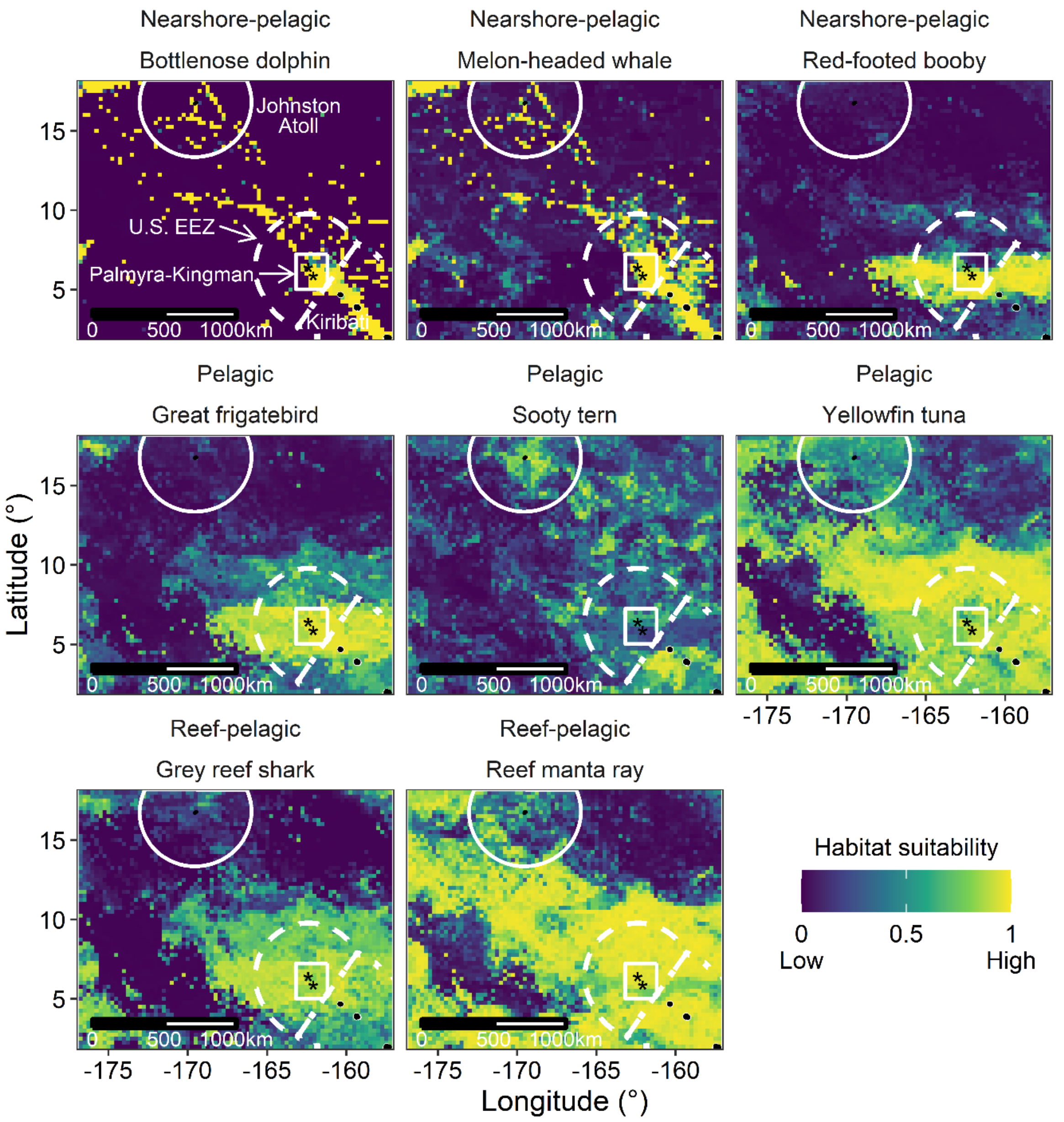

Figure S3: Habitat suitability calculated per species per 0.25 x 0.25° grid cell, predicted for boreal spring/summer (June 1, 2022). The relative influence of variables per species are listed in Table 3. Habitat suitability is indicated by gradient scale of dark–light colors that indicate low to high habitat suitability, respectively. Habitat suitability was predicted by species distribution models that were estimated separately for each species via boosted regression trees that used background sampling. Solid white lines indicate boundaries of the Pacific Islands Heritage Marine National Monument, dashed lines indicate the U.S. Exclusive Economic Zone (EEZ), and dotted lines indicate the Kiribati EEZ. Geographic features are labeled in the top-left panel. Species are grouped into habitats (top rows of text). Map lines delineate study areas and do not necessarily depict accepted national boundaries.

Figure S4

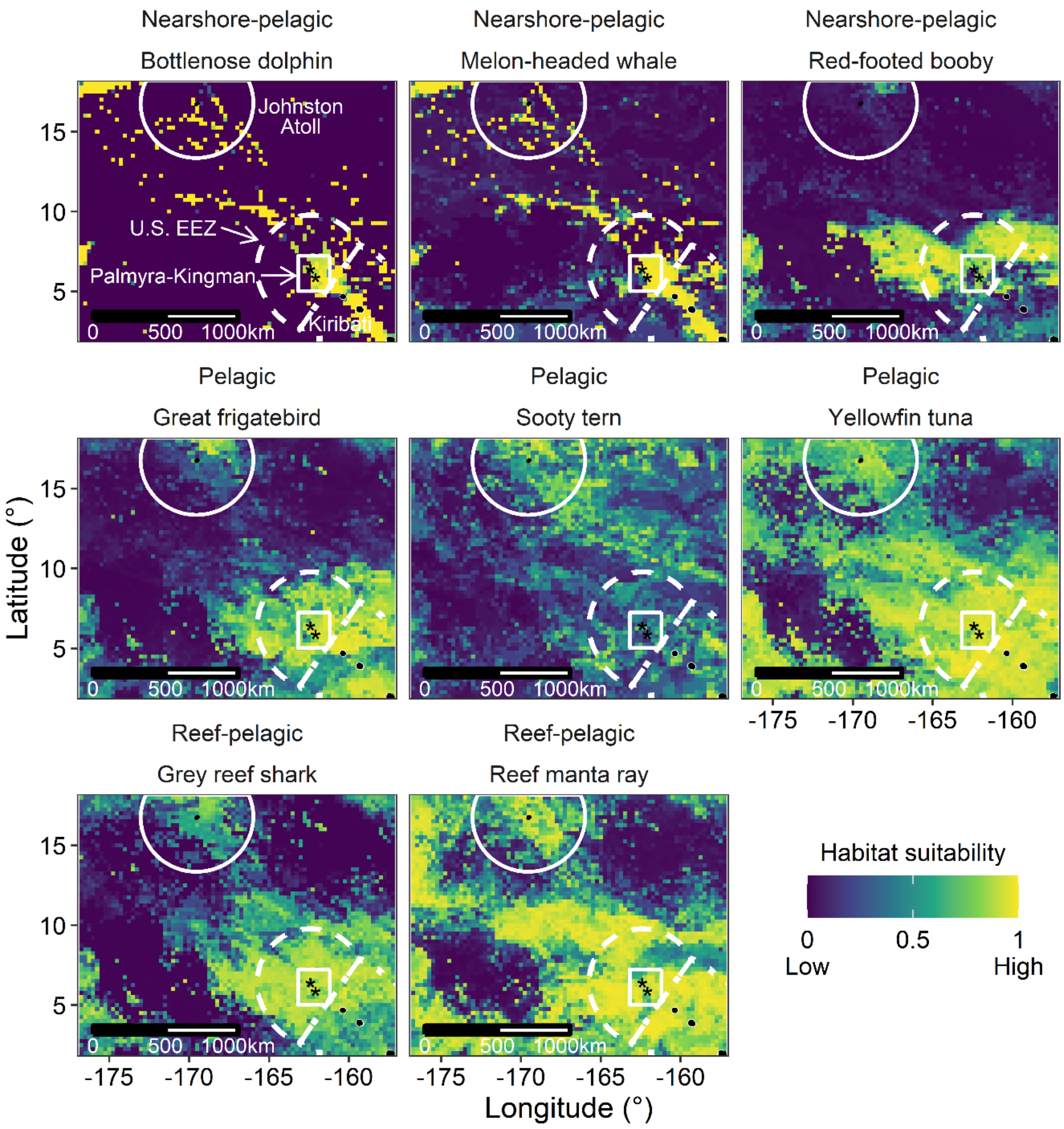

Figure S4: Habitat suitability per species predicted for boreal winter (December 1, 2022). The relative influence of variables per species are listed in Table 3. Habitat suitability is indicated by gradient scale of dark–light colors that indicate low to high habitat suitability, respectively. Habitat suitability was predicted by species distribution models that were estimated separately for each species via boosted regression trees that used background sampling. Solid black lines indicate Pacific Islands Heritage Marine National Monument boundaries and dashed lines indicate the U.S. Exclusive Economic Zone. Boundaries are labeled in the top-left box to provide geographical context. Species are grouped into primary habitat types (top). Map lines delineate study areas and do not necessarily depict accepted national boundaries.

Figure S5

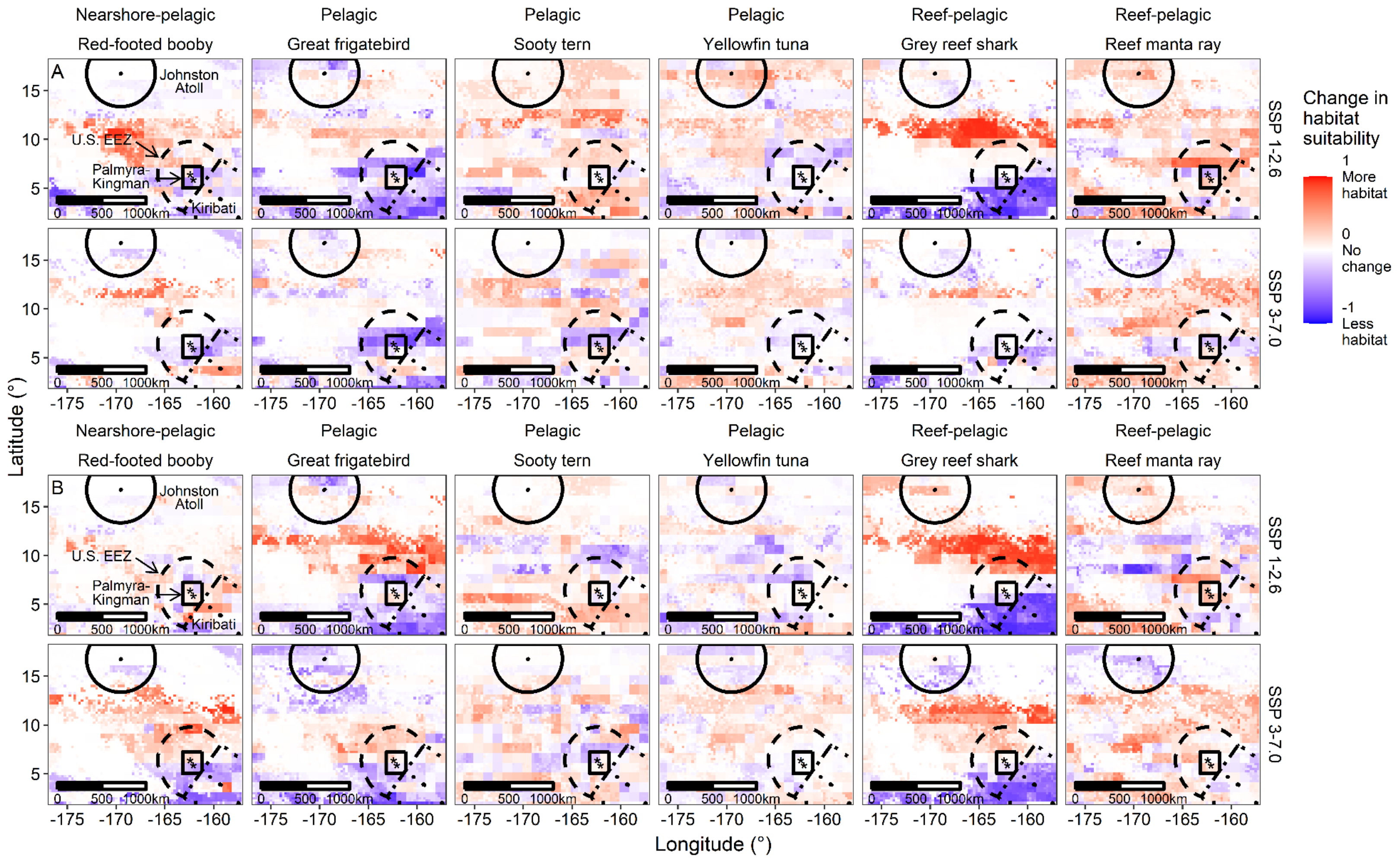

Figure S5: Predicted change in habitat suitability under two climate scenarios, SSP 1-2.6 and SSP 3-7.0, and two time periods, 2040–2050 (**A**) and 2090–2100 (**B**) based on the boreal summer model (June 1, 2022). The scale of change ranges from -1 (decreased habitat; blue colors) to 1 (increased habitat; red colors). For regional orientation and geographical context, features are labeled for red-footed boobies (upper left panels)

within each decadal plot group. Median habitat suitability values and the percent change in habitat suitability are quantified in Tables S6 and S7. Map lines delineate study areas and do not necessarily depict accepted national boundaries.

Figure S6

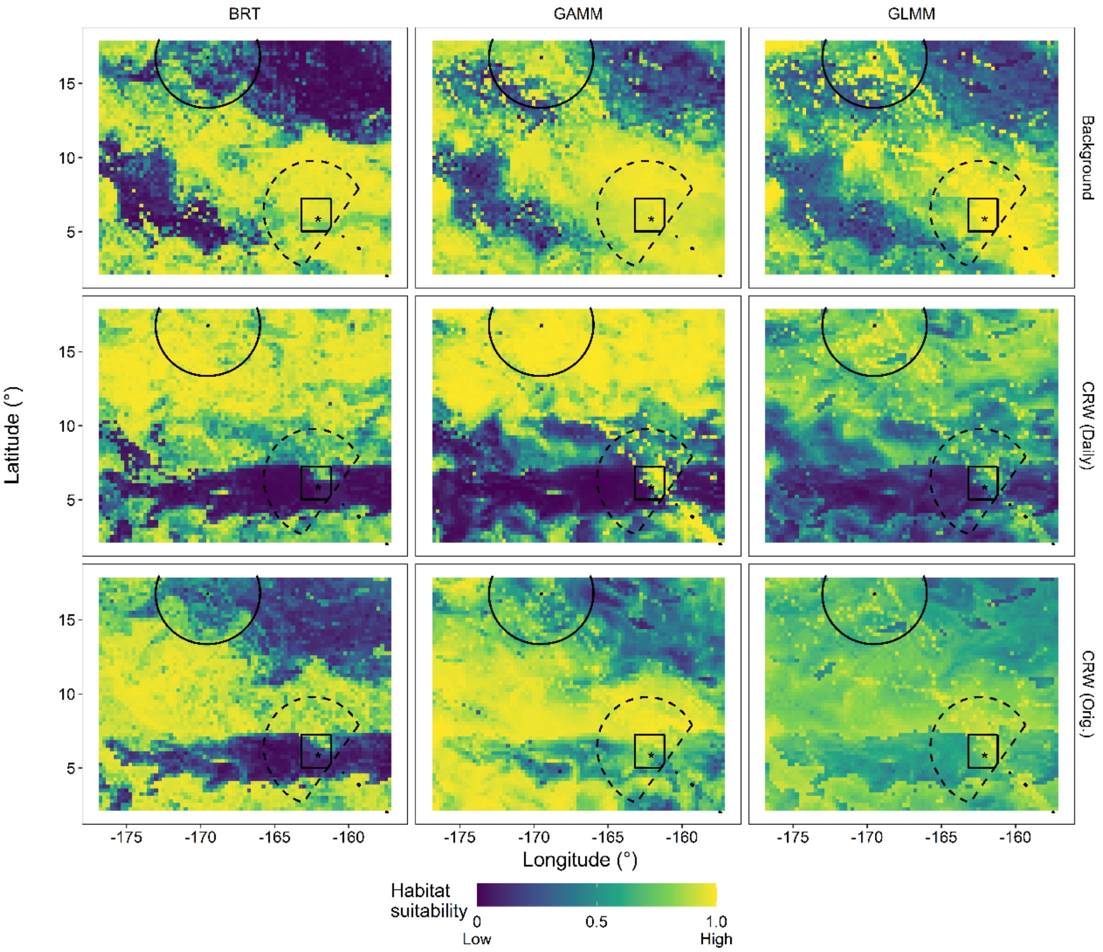

Figure S6: **Species distribution model results for reef manta ray**. Model types correspond to columns and pseudo-absence types correspond to rows. Abbreviations: BRT: boosted regression tree; GAMM: generalized additive mixed model; GLMM: generalized linear mixed model; CRW: correlated random walk. CRW are presented for two temporal resolutions: daily (CRW was conducted on median daily locations per individual) and original (CRW was conducted at the original tag sample resolution per individual, and then the median daily location was calculated). Black polygons represent Pacific Islands Heritage Marine National Monument boundaries, dashed black line represents the U.S. exclusive economic zone surrounding the Palmyra-Kingman unit, and asterisk indicates the location of Palmyra Atoll. Map lines delineate study areas and do not necessarily depict accepted national boundaries.

Figure S7

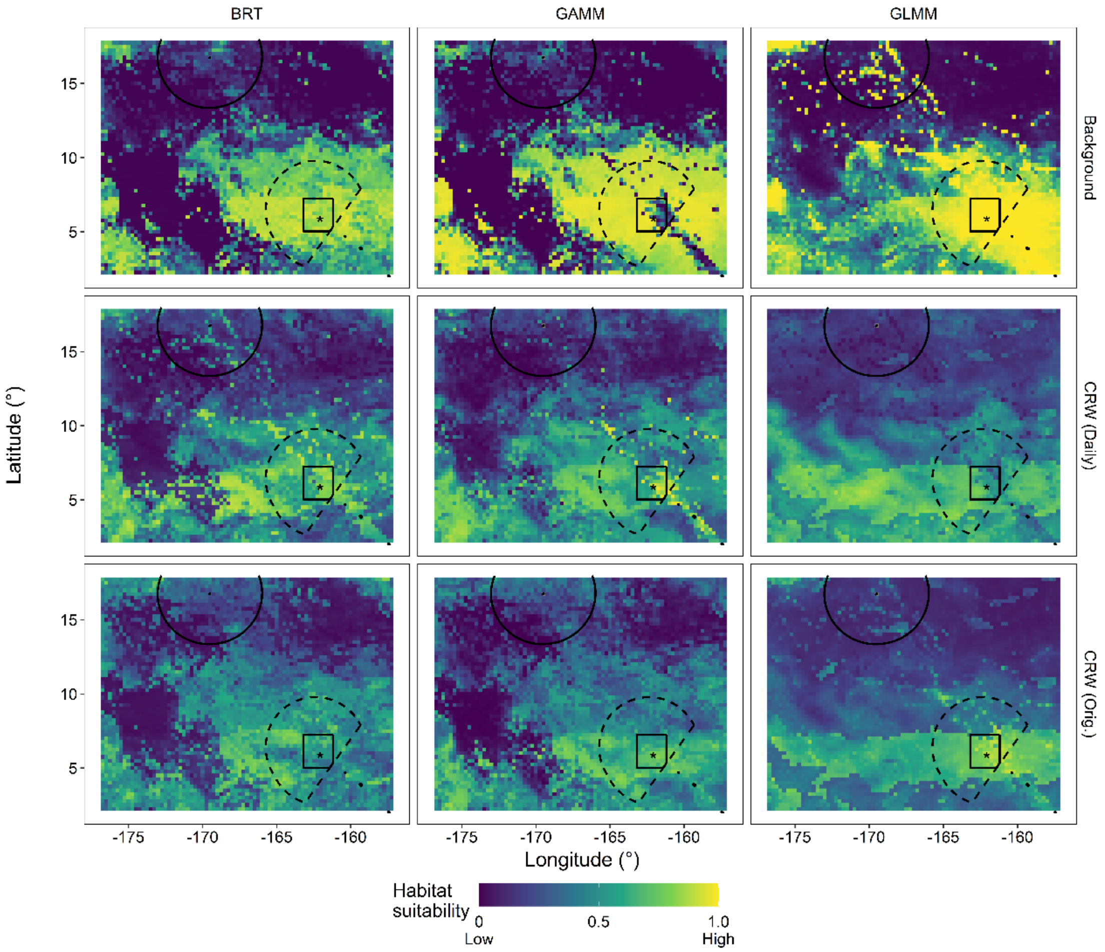

Figure S7: **Species distribution model results for grey reef shark**. Figure legend and abbreviations are the same as in Figure S6. Map lines delineate study areas and do not necessarily depict accepted national boundaries.

Figure S8

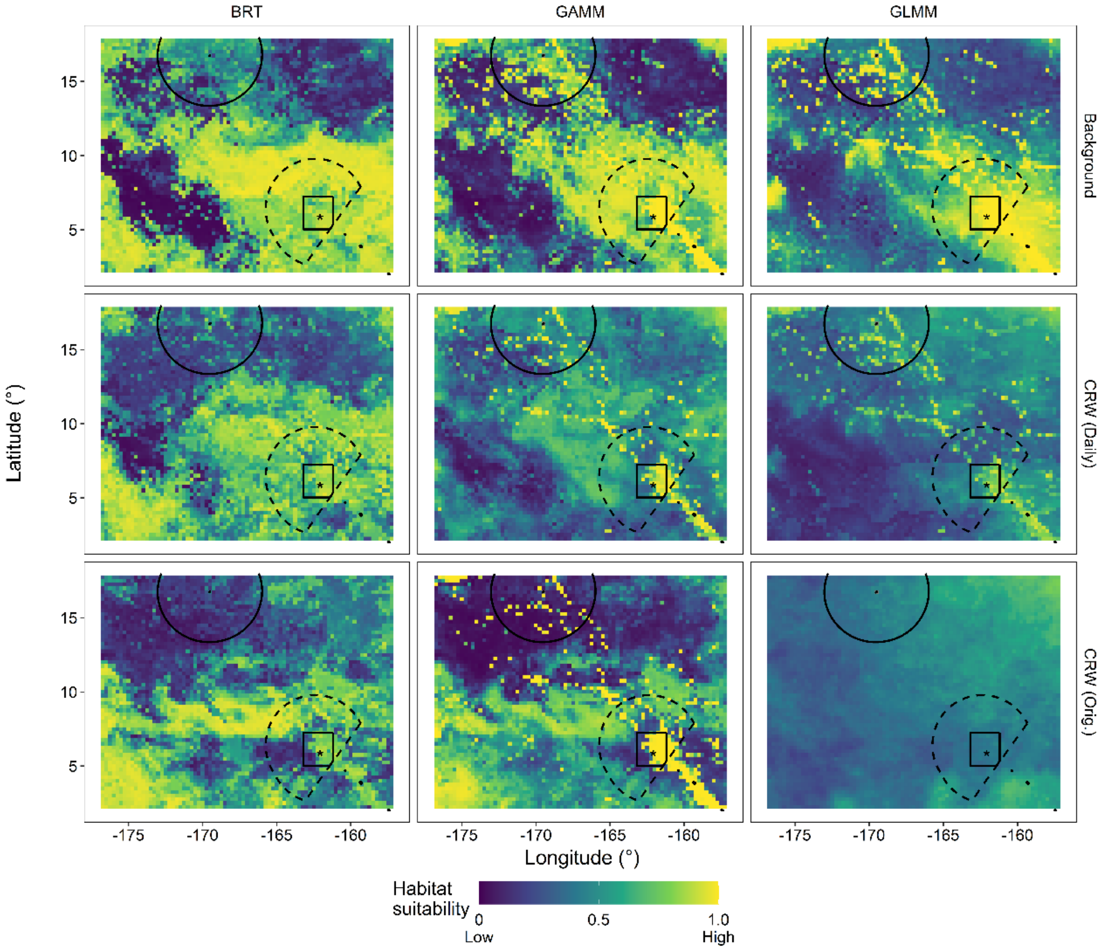

Figure S8: **Species distribution model results for yellowfin tuna**. Figure legend and abbreviations are the same as in Figure S6. Map lines delineate study areas and do not necessarily depict accepted national boundaries.

Figure S9

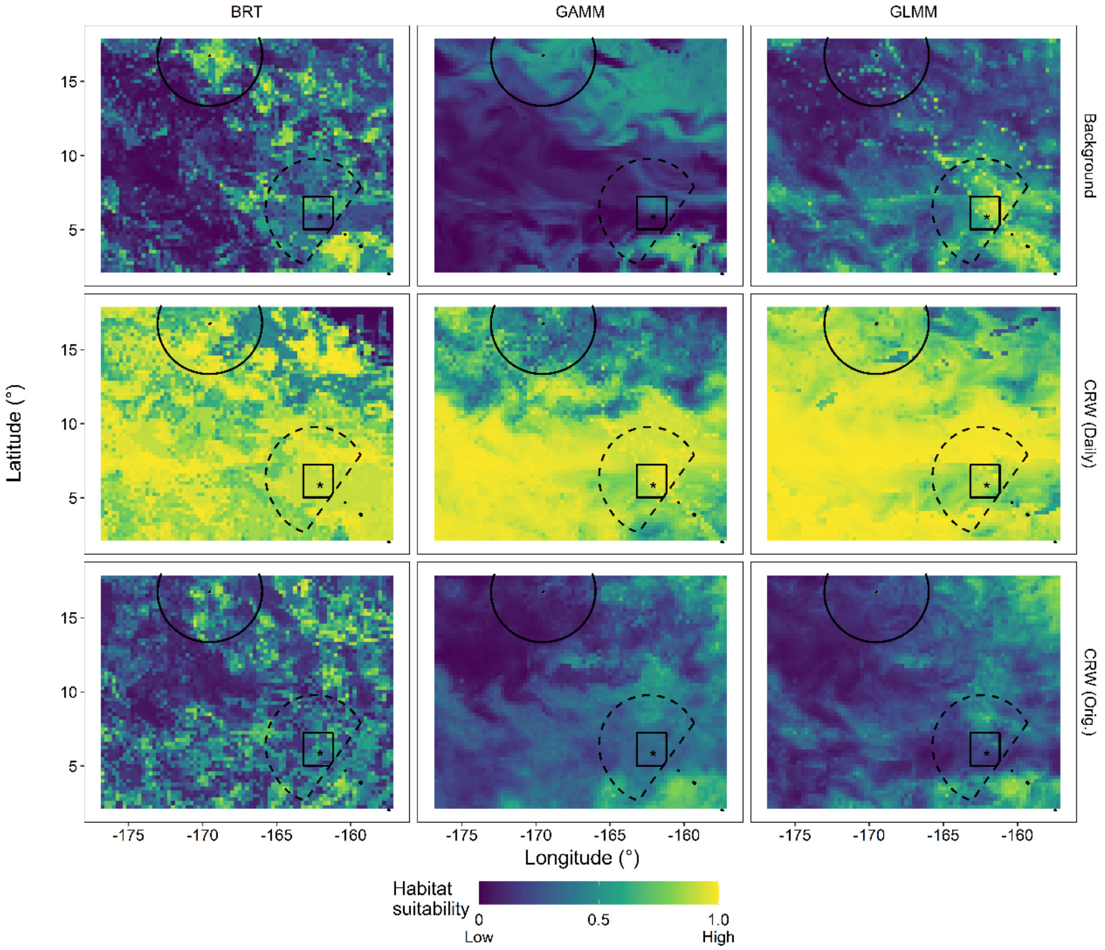

Figure S9: **Species distribution model results for sooty tern**. Figure legend and abbreviations are the same as in Figure S6. Map lines delineate study areas and do not necessarily depict accepted national boundaries.

Figure S10

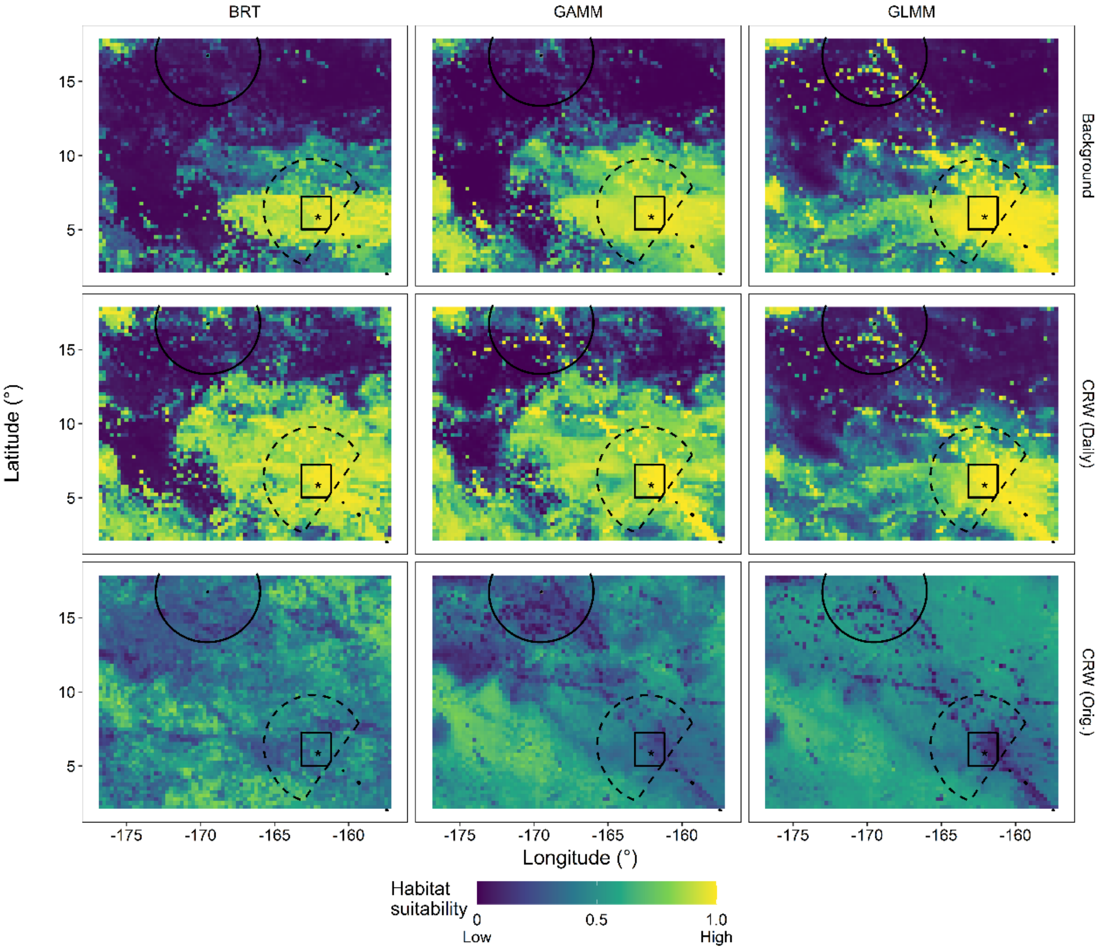

Figure S10: **Species distribution model results for great frigatebird**. Figure legend and abbreviations are the same as in Figure S6. Map lines delineate study areas and do not necessarily depict accepted national boundaries.

Figure S11

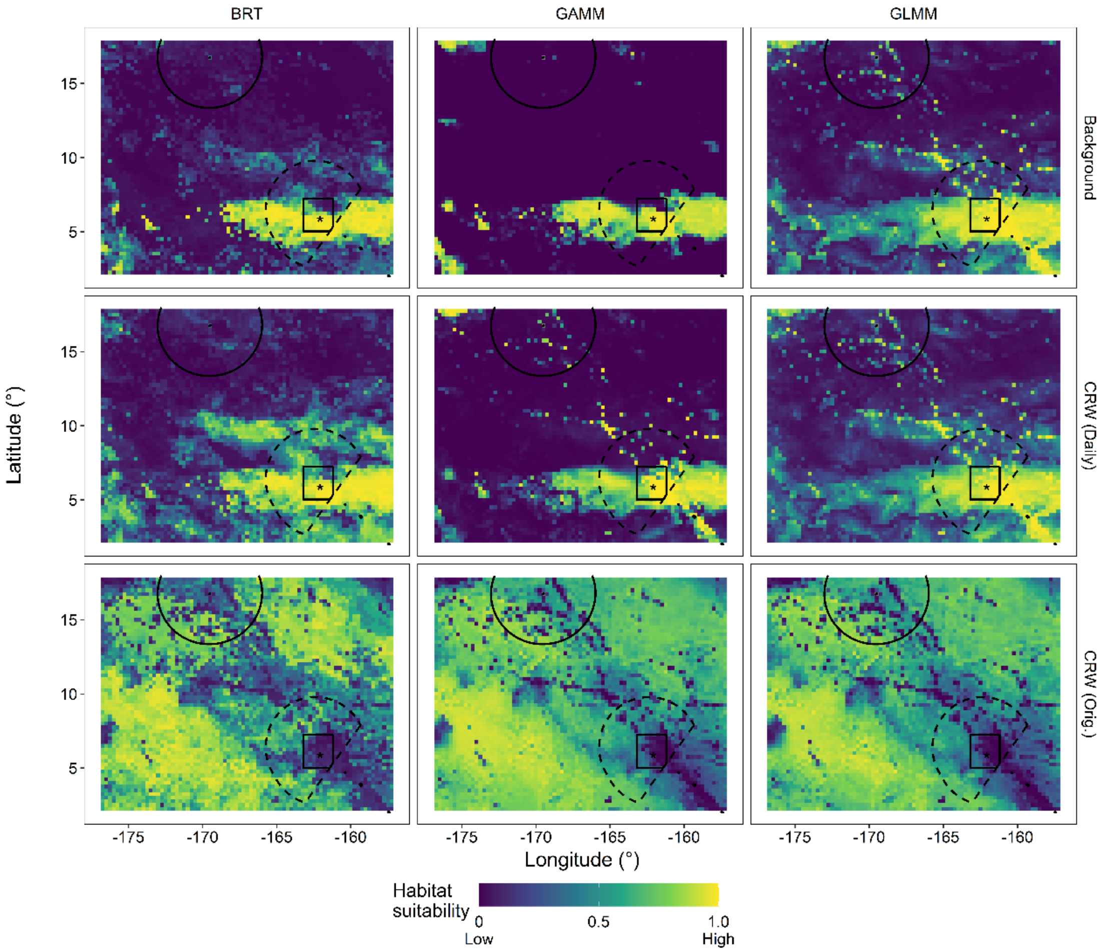

Figure S11: **Species distribution model results for red-footed booby**. Figure legend and abbreviations are the same as in Figure S6. Map lines delineate study areas and do not necessarily depict accepted national boundaries.

Figure S12

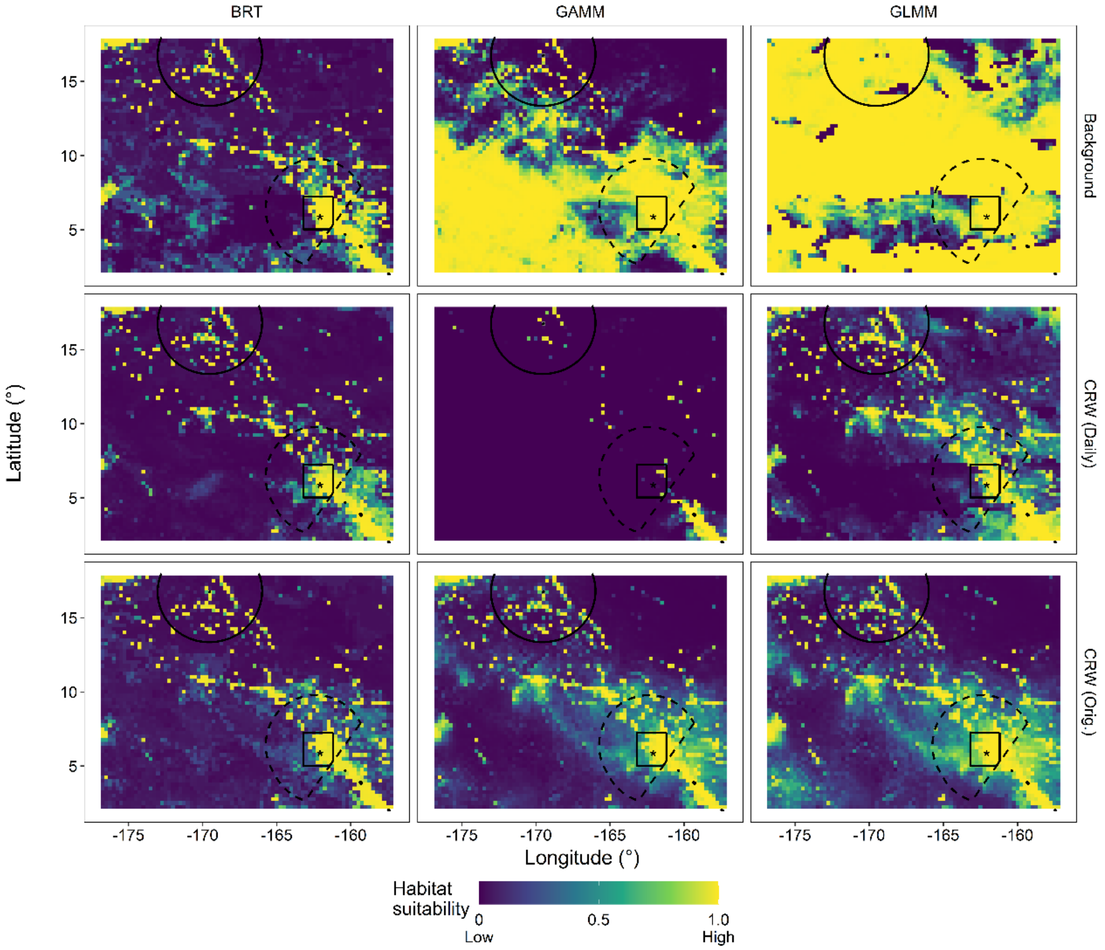

Figure S12: **Species distribution model results for melon-headed whale**. Figure legend and abbreviations are the same as in Figure S6. Map lines delineate study areas and do not necessarily depict accepted national boundaries.

Figure S13

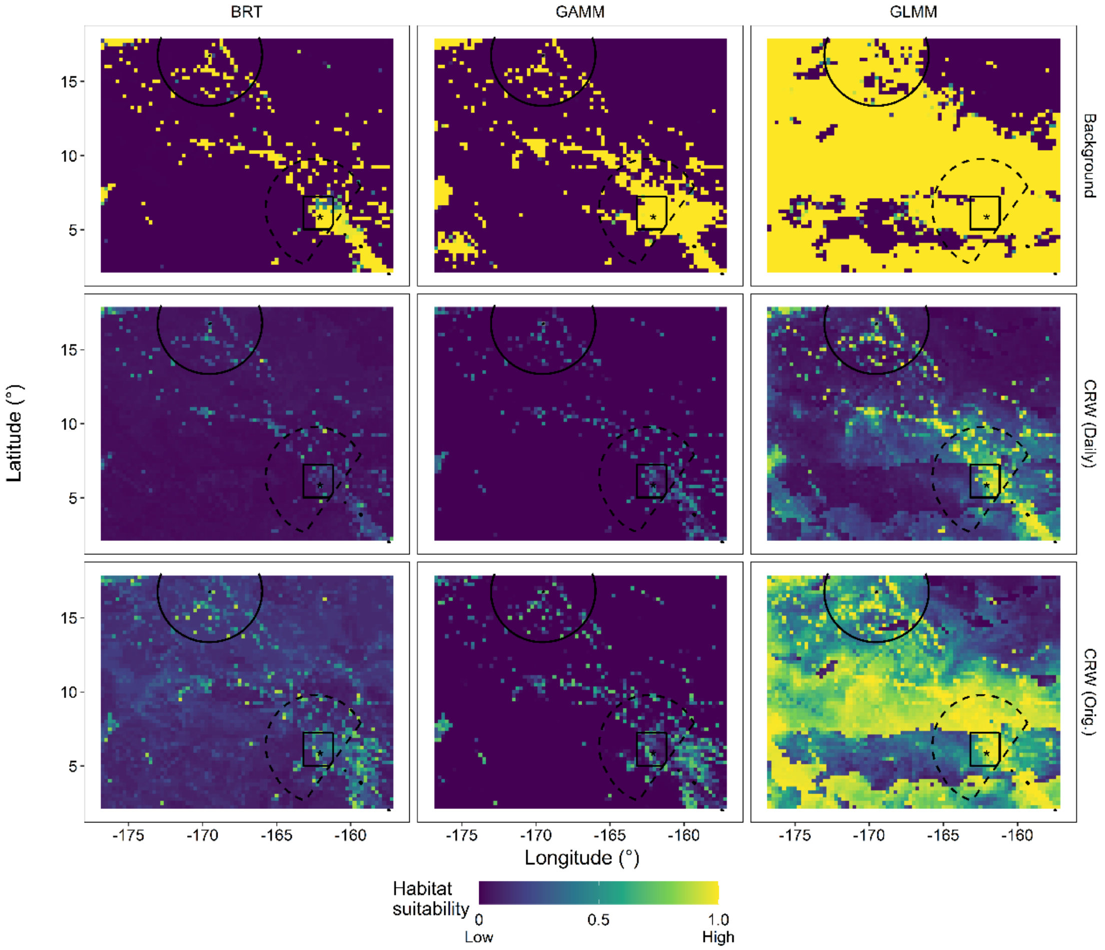

Figure S13: **Species distribution model results for bottlenose dolphin**. Figure legend and abbreviations are the same as in Figure S6. Map lines delineate study areas and do not necessarily depict accepted national boundaries.
